# Cystine deprivation triggers CD36-mediated ferroptosis and dysfunction of tumor infiltrating CD8^+^ T cells

**DOI:** 10.1038/s41419-024-06503-1

**Published:** 2024-02-15

**Authors:** Chenfeng Han, Minmin Ge, Pengfei Xing, Tian Xia, Cangang Zhang, Kaili Ma, Yifu Ma, Shicheng Li, Wenhui Li, Xiaowei Liu, Baojun Zhang, Liyuan Zhang, Lianjun Zhang

**Affiliations:** 1 National Key Laboratory of Immunity and Inflammation, Suzhou Institute of Systems Medicine, Chinese Academy of Medical Sciences & Peking Union Medical College, Suzhou, Jiangsu 215123 China; 2 Key Laboratory of Synthetic Biology Regulatory Element, Institute of Systems Medicine, Chinese Academy of Medical Sciences and Peking Union Medical College, Suzhou, Jiangsu 215123 China; 3 Department of Radiotherapy & Oncology, The Second Affiliated Hospital of Soochow University, Suzhou, China; 4 Center for Cancer Diagnosis and Treatment, The Second Affiliated Hospital of Soochow University, Suzhou, China; 5 Laboratory for Combined Radiotherapy and Immunotherapy of Cancer, The Second Affiliated Hospital of Soochow University, Suzhou, China; 6 Institute of Biology and Medical Sciences (IBMS), Soochow University, Suzhou, Jiangsu 215123 China; 7 Department of Pathogenic Microbiology and Immunology, School of Basic Medical Sciences, Xi’an Jiaotong University, Xi’an, Shaanxi China; 8 Institute of Infection and Immunity, Translational Medicine Institute, Xi’an Jiaotong University Health Science Center, Xi’an, Shaanxi China; 9 Key Laboratory of Environment and Genes Related to Diseases, Xi’an Jiaotong University, Xi’an, Shaanxi China; 10 Xi’an Key Laboratory of Immune Related Diseases, Xi’an, Shaanxi China

**Keywords:** Tumour immunology, T cells

## Abstract

Cancer cells develop multiple strategies to evade T cell-mediated killing. On one hand, cancer cells may preferentially rely on certain amino acids for rapid growth and metastasis. On the other hand, sufficient nutrient availability and uptake are necessary for mounting an effective T cell anti-tumor response in the tumor microenvironment (TME). Here we demonstrate that tumor cells outcompete T cells for cystine uptake due to high Slc7a11 expression. This competition induces T-cell exhaustion and ferroptosis, characterized by diminished memory formation and cytokine secretion, increased PD-1 and TIM-3 expression, as well as intracellular oxidative stress and lipid-peroxide accumulation. Importantly, either Slc7a11 deletion in tumor cells or intratumoral cystine supplementation improves T cell anti-tumor immunity. Mechanistically, cystine deprivation in T cells disrupts glutathione synthesis, but promotes CD36 mediated lipid uptake due to dysregulated cystine/glutamate exchange. Moreover, enforced expression of glutamate-cysteine ligase catalytic subunit (Gclc) promotes glutathione synthesis and prevents CD36 upregulation, thus boosting T cell anti-tumor immunity. Our findings reveal cystine as an intracellular metabolic checkpoint that orchestrates T-cell survival and differentiation, and highlight Gclc as a potential therapeutic target for enhancing T cell anti-tumor function.

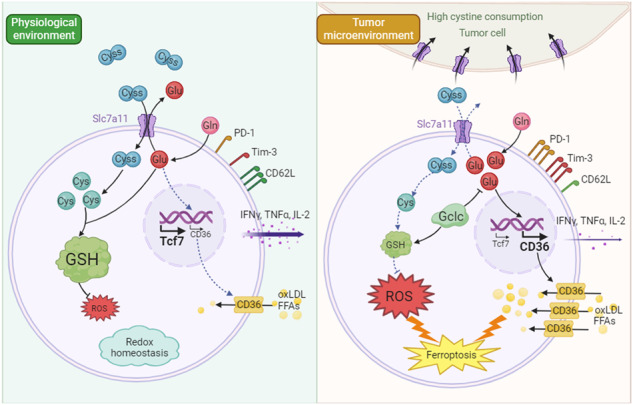

## Introduction

Adoptive T-cell transfer and immune checkpoint blockade have achieved durable clinical outcomes in a certain fraction of cancer patients [1, 2], but the majority of patients do not benefit from those treatments due to the highly immunosuppressive tumor microenvironment (TME), which poses a major challenge for effective immunotherapies against solid tumors [3–5]. In the TME, multiple factors contribute to immune suppression, including low oxygen and pH levels, limited nutrient availability (e.g., glucose, fatty acids, and amino acids), accumulation of immunosuppressive metabolites (e.g., ROS, lactate, and lipids), and increased secretion of immunosuppressive cytokines as well as chemokines [6–8]. In this regard, T cells gradually lose the ability of memory or stemness characteristics, and undergo differentiation towards exhaustion and dysfunction. This is characterized by diminished expression of TCF1, LY108 and CD62L, along with elevated levels of exhaustion markers such as PD-1, TIM-3, and TOX [9–11].

Recently, mounting evidence indicates that the limited amino acid availability in the TME profoundly shapes T-cell functionality [12–14]. Among conditionally essential amino acids, cystine has emerged as a notably scarce amino acid in the TME due to its high consumption by both tumor cells and immunosuppressive cells [15–17]. It is known that cystine and its reduced form of cysteine are essential for T-cell proliferation, DNA and protein synthesis, and cytokine secretion [18–20]. Beyond supporting growth and metabolic demands, cystine also exerts antioxidative properties [21, 22], shielding T cells from oxidative damage in the TME. Despite several studies have reported decreased cystine levels in the TME [17, 23], the impact of reduced cystine on the anti-tumor capabilities of cytotoxic T lymphocytes remains unclear. We hypothesize that T cells may fail to mount a productive anti-tumor response under the setting of cystine deprivation. Therefore, a comprehensive investigation is warranted to explore the effects and mechanisms of cystine deprivation on the survival, differentiation, and functionality of cytotoxic T lymphocytes.

In extracellular space, cysteine primarily exists in its oxidized form cystine [13]. System Xc^-^ transporter, consisting of Slc7a11 and Slc3a2 subunits, is responsible for the uptake of extracellular cystine in exchange for glutamate [24]. Once transported into cells, cystine is immediately reduced to cysteine [13], which is the rate-limiting amino acid for glutathione synthesis to maintain redox homeostasis [21, 22]. Oxidative stress, Fe^2+^ and lipid accumulation are key factors that contribute to ferroptosis. Cystine deprivation-induced ferroptosis in tumor cells has been extensively explored [25]. In this regard, cystine deprivation impedes the generation of glutathione, co-enzyme A and iron-sulfur clusters, causing increased lipid peroxidation and Fe^2+^ uptake [26–28]. In addition, cystine deprivation dampens the mTOR/GPX4 signaling axis, suppressing the clearance of lipid peroxides [29]. Notably, cystine deficiency-induced glutamate accumulation has been identified as a pivotal driver of reactive oxygen species (ROS) production and ferroptosis in tumor cells [30–32]. Nevertheless, it remains unclear whether cystine starvation in the TME triggers T-cell ferroptosis and impairs anti-tumor immunity.

In this study, we demonstrated that CD8^+^ T cells failed to outcompete tumor cells for cystine uptake, leading to cystine starvation. Cystine deprivation induced oxidative stress and glutamate accumulation, consequently upregulating CD36-mediated lipid uptake. This process led to T-cell exhaustion and ferroptosis, characterized by reduced TCF1, LY108 and CD62L expression, accompanied by increased PD-1 and TIM-3 expression, elevated oxidative stress, and lipid accumulation. Additionally, cytokine secretion, including IFNγ, TNFα, and IL-2, was declined in cystine-deprived T cells. Notably, to improve the anti-tumor capacity of T cells under cystine starvation, we engineered T cells expressing the rate-limiting enzyme of glutathione synthesis, Gclc. Gclc, a crucial regulator of intracellular antioxidants, has recently been reported to counteract ferroptosis by consuming glutamate in cystine-deficient tumor cells [33]. These observations imply the potential of Gclc in safeguarding T-cell survival and function under the cystine-deprived TME. Importantly, Gclc overexpression boosts T cell anti-tumor functionality via glutathione synthesis and glutamate consumption. Thus, our findings unveil a novel mechanism of tumor-induced immunosuppression within the TME, and emphasize the therapeutic potential of modulating cystine availability to fuel T cell anti-tumor immunity.

## Results

### Tumor cells outcompete T cells for cystine uptake, leading to T-cell exhaustion and death

**Fig. 1:**
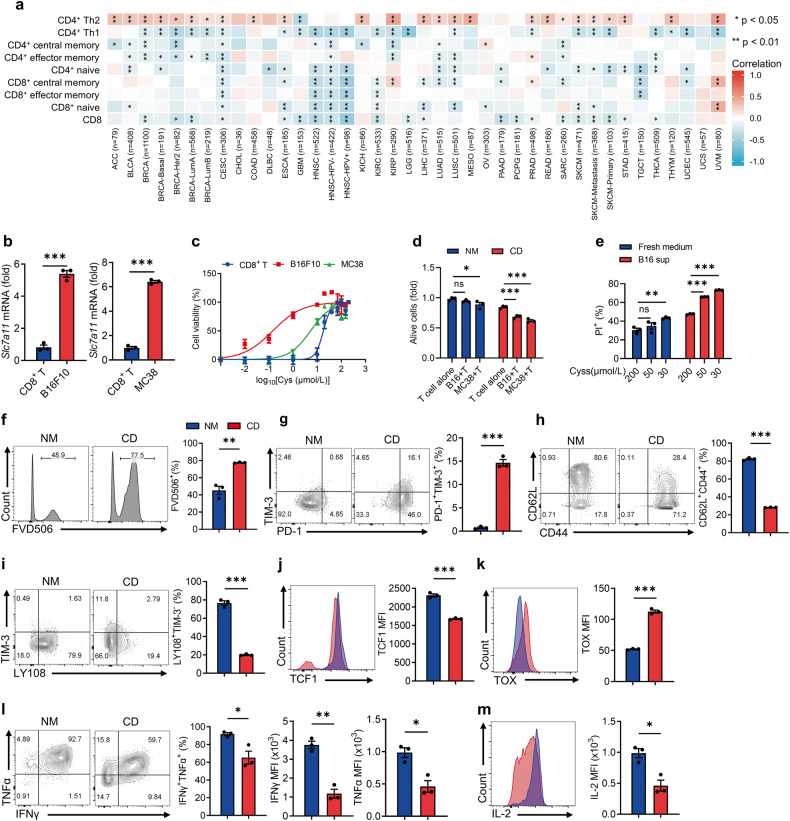
Competitive uptake of cystine by tumor cells leads to T-cell exhaustion and death. **a** Analysis of the correlation between immune infiltration and *SLC7A11* expression in tumors via TIMER 2.0 database. **b** *Slc7a11* mRNA expression in CD8^+^ T effector cells and tumor cells (*n* = 3 per group). **c** CD8^+^ T, B16F10, and MC38 cells were cultured with varying cystine concentrations, and cell viability was detected by CCK8 assay (*n* = 3 per group, CD8^+^ T EC50 = 18.05, B16F10 EC50 = 0.1444, MC38 EC50 = 5.326). **d** CD8^+^ T cells and B16F10 cells were co-cultured at 1:1 for 24 h in NM and CD, and cell viability was detected by CCK8 assay (*n* = 3 per group). **e** CD8^+^ T cells were cultured for 24 h in fresh medium or B16F10 cell supernatant with varying cystine concentrations, and the percentages of dead cells were detected by flow cytometry (*n* = 3 per group). **f**–**m** Effects of prolonged cystine starvation on CD8^+^ T-cell differentiation and death. T cells were cultured in NM or CD for 72 h (*n* = 3 per group), and the percentages of dead cells (**f**), PD-1^+^TIM-3^+^ subset (**g**), CD62L^+^CD44^+^ subset (**h**), LY108^+^TIM-3^-^ subset (**i**), and the levels of TCF1 (**j**) and TOX (**k**) were detected by flow cytometry. IFNγ, TNFα (**l**), and IL-2 (**m**) secretion was measured after re-stimulation. Each symbol represents one individual. Data are presented as mean ± s.e.m. *p* values are measured by two-tailed unpaired Student’s *t* test (**b**, **f**–**m**) and one-way ANOVA with Tukey’s multiple comparison test (**d**, **e**). *p < 0.05, **p < 0.01, ***p < 0.001.

Firstly, we compared the capability of cystine uptake between T cells and tumor cells, confirming that *SLC7A11*, the functional subunit of system Xc-, was upregulated in various human tumors (Supplementary Fig. 1a). Consistent with previous studies [17, 23], we noted a significant decrease of cystine concentration in the mouse tumor-interstitial fluid compared to the serum (Supplementary Fig. 1b). Notably, a negative correlation was observed between tumor *SLC7A11* expression and T-cell infiltration, while *SLC7A11* expression was positively associated with immunosuppressive Th2 infiltrates (Fig. 1a), suggesting that elevated cystine uptake by tumor cells might hamper the anti-tumor immunity. As CD8^+^ T cells plays a pivotal role in anti-tumor immune responses [34], we thus focused on their response to restricted cystine due to high consumption by tumor cells. Despite CD8^+^ T cells up-regulating Slc7a11 expression under cystine starvation (Supplementary Fig. 1c, d), it is worth noting that *Slc7a11* expression in tumor-infiltrated CD8^+^ T cells was significantly lower than that in tumor cells (Fig. 1b). These observations suggest that CD8^+^ T cells may suffer from incompetent uptake of cystine within the TME.

Next, we assessed the half-maximal effective concentration (EC50) of cystine for maintaining CD8^+^ T and tumor cell viability. Notably, CD8^+^ T cells were more sensitive to cystine starvation than tumor cells (Fig. 1c). To investigate the impacts of cystine starvation on T cells in vitro, T cells were cultured in normal medium (NM, 200 μmol/L cystine) and cystine-deprived medium (CD, 20 μmol/L cystine), unless otherwise specified. Using a co-culture system (Supplementary Fig. 1f), we co-cultured T cells and tumor cells in a 1:1 ratio in both NM and CD for 24 h. Under cystine deprivation, we observed evident T-cell death, whereas T cells stayed alive under sufficient cystine (Fig. 1d). Additionally, T cells were cultured with either fresh medium or tumor cell supernatant with varying cystine concentrations for 24 h. Regardless of cystine concentrations, fresh medium minimally affected T-cell survival, while tumor-cell supernatant significantly induced T-cell death when the culture medium contained less than 50 μmol/L cystine (Fig. 1e and Supplementary Fig. 1e). These findings indicated that cystine consumption by tumor cells impaired CD8^+^ T-cell survival.

To further explore the impacts of prolonged cystine starvation on T cells, activated T cells were cultured under cystine deprivation for 72 h (Supplementary Fig. 1c), and we noted a significant increase in T-cell death (Fig. 1f). Concomitantly, we observed an increased frequency of PD-1^+^TIM-3^+^ terminal exhausted T (Tex) cells in cystine-deprived T cells, along with decreased CD62L^+^CD44^+^ central memory T (Tcm) and LY108^+^TIM-3^-^ progenitor exhausted populations (Fig. 1g–i). Moreover, cystine starvation significantly reduced TCF1 expression while enhancing TOX expression in T cells (Fig. 1j, k). Furthermore, long-term cystine deprivation impeded T cell cytokine-secreting capacity (Fig. 1l, m). These findings indicated that cystine deprivation induces T-cell exhaustion and death, hampering their effector function.

### Cystine deprivation results in T-cell ferroptosis and terminal exhaustion

**Fig. 2:**
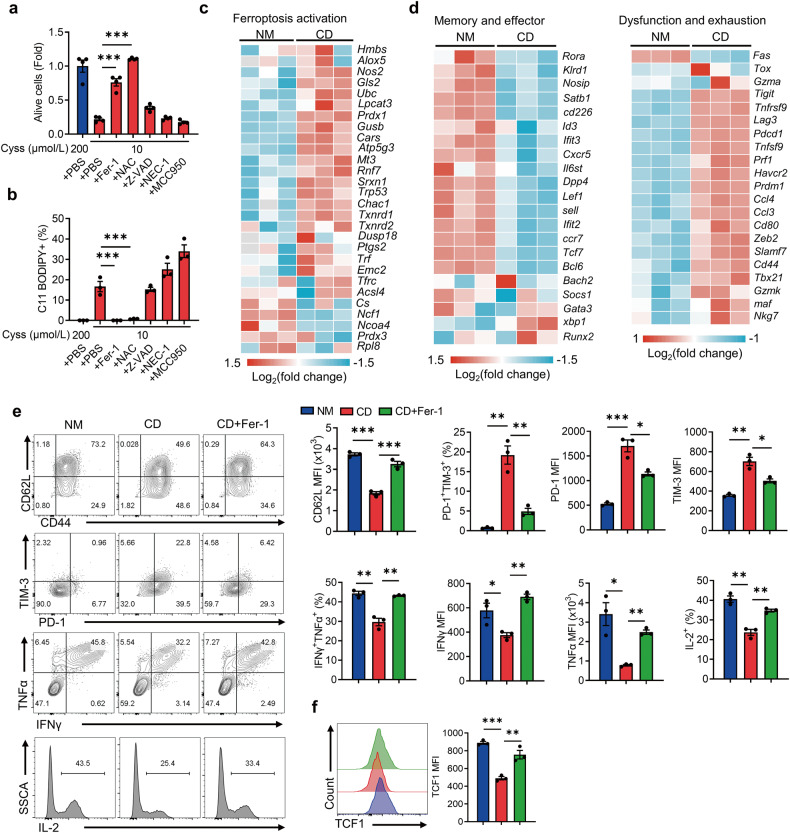
Cystine deprivation triggers evident ferroptosis and exhaustion of CD8^+^ T cells. **a**, **b** T cells were cultured in 10 μmol/L cystine medium containing Fer-1, NAC, Z-VAD, NEC-1, and MCC950, respectively (*n* = 3 per group). Cell viability was detected via CCK8 assay (**a**), and lipid peroxidation was measured via C11 BODIPY staining (**b**). **c**, **d** RNA-seq analysis of ferroptosis activation-related genes, memory/effector-related genes and dysfunction/exhaustion-related genes of T cells cultured in NM or CD (*n* = 3 per group). **e**, **f** Flow cytometry analysis of CD62L expression, PD-1^+^TIM-3^+^ T-cell subset, cytokine secretion (**e**) and TCF1 expression (**f**) of the indicated T cells (*n* = 3 per group). Each symbol represents one individual. Data are presented as mean ± s.e.m. *p* values are measured by one-way ANOVA with Tukey’s multiple comparison test. *p < 0.05, **p < 0.01, ***p < 0.001.

To further explore how cystine deprivation impacts T-cell survival and differentiation, we employed a large panel of small molecule inhibitors of various cell death pathway and determined whether they could rescue the survival defects of cystine-deprived T cells. For instance, Ferrostatin-1 (Fer-1) acts as a synthetic antioxidant mitigating lipid peroxides to prevent ferroptosis [35]. Z-VAD-FMK (Z-VAD) inhibits caspase activity to prevent apoptosis [36]. Necrostatin-1 (NEC-1) suppress necroptosis by inhibiting receptor-interacting protein kinase 1 activity [37]. MCC950 inhibits the activation of the NOD-like receptor protein 3, preventing inflammasome formation and pyroptosis [38]. Notably, Fer-1 significantly preserved T-cell survival and prevented lipid peroxidation (Fig. 2a, b, and Supplementary Fig. 2a), indicating T-cell death under cystine deprivation was mainly due to increased ferroptosis. In addition, administration of N-acetyl cysteine (NAC, a cell-permeable analog of cysteine) further indicated that cysteine supplementation restored cystine deprived T-cell survival. Besides, RNA sequencing analysis confirmed elevated expression of genes associated with lipid peroxidation and ferroptosis in cystine-deprived T cells, with no significant change in ferroptosis inhibition-related genes compared to normal medium-cultured T cells (Fig. 2c and Supplementary Fig. 2c). Additionally, we observed increased expression of dysfunction and exhaustion-related signature genes while memory and effector-related gene expression was decreased in cystine-deprived T cells (Fig. 2d). These data corroborate that cystine deprivation induces T-cell ferroptosis and terminal exhaustion. Considering the significantly decreased cystine content at tumor sites compared to normal tissues [17, 23], we verified increased ferroptosis of tumor-infiltrating CD8^+^ T cells (Supplementary Fig. 2b), implying that cystine deprivation in the TME may indeed trigger T-cell ferroptosis. Furthermore, Fer-1 and NAC increased the Tcm population, reduced the Tex subset (Fig. 2e and Supplementary Fig. 2d), restored the TCF1 expression (Fig. 2f and Supplementary Fig. 2e), and improved T cell cytokine-secretion capacity (Fig. 2e and Supplementary Fig. 2d). Collectively, our observations support that inhibiting ferroptosis effectively alleviates T-cell exhaustion and prevents cell death induced by cystine starvation.

### Restricted cystine uptake by tumor cells reduces T-cell ferroptosis and restores anti-tumor immunity

**Fig. 3:**
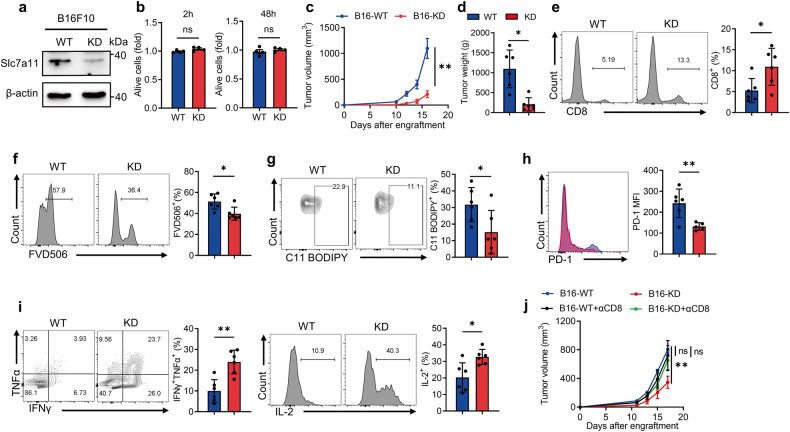
Inhibition of cystine uptake by tumor cells alleviates ferroptosis of CD8^+^ T cells and enhances anti-tumor immunity. **a** Western blot analysis of Slc7a11 expression in WT and Slc7a11-KD B16F10 cells. **b** CCK8 assay measuring cell viability of WT and KD B16F10 cells after 2 h and 48 h in vitro culturing (*n* = 4 per group). **c**, **d** Tumor volume (**c**) and tumor weight (**d**) of WT and KD B16F10 tumors (*n* = 6 per group). **e** Flow cytometry measuring the percentages of tumor-infiltrating CD8^+^ T cells in WT and KD tumors. **f** The percentages of dead CD8^+^ T cells in WT and KD tumors. **g** Lipid peroxidation of CD8^+^ T cells in WT and KD tumors. **h** PD-1 expression of CD8^+^ T cells in WT and KD tumors (one outlier was removed from KD). **i** Levels of IFNγ, TNFα, and IL-2 secretion of the indicated T cells. **j** Tumor volume of WT and KD tumors after CD8 antibody administration (WT: *n* = 4; KO: *n* = 5; WT + αCD8: *n* = 5; KD + αCD8: *n* = 6). Each symbol represents one individual. Data are mean ± s.e.m. *p* values are measured by two-tailed unpaired Student’s *t* test. Ns not significant, *p < 0.05, **p < 0.01.

To explore the impact of impeding tumor competition for cystine on T cell anti-tumor immunity, we generated Slc7a11 knockdown (KD) B16F10 cells (Fig. 3a and Supplementary Fig. 3a). Of note, Slc7a11-KD did not impede tumor cell growth in vitro (Fig. 3b), but significantly suppressed tumor growth in immunocompetent mice (Fig. 3c, d, and Supplementary Fig. 3b). In Slc7a11-KD tumors, the tumor-infiltrating CD8^+^ T cells was markedly increased (Fig. 3e), accompanied by a reduction in CD8^+^ T-cell ferroptosis (Fig. 3f, g). Moreover, intratumoral T cells displayed reduced exhaustion phenotype upon Slc7a11-KD in B16F10 cells, along with enhanced cytokine secretion (Fig. 3h, i). Nevertheless, Slc7a11-KD in tumor cells had little impact on CD8^+^ T cells in draining lymph nodes (DLN) and spleens (Supplementary Fig. 3c–h). To confirm that the restricted tumor progression of Slc7a11-KD tumor cells mainly relies on improved CD8^+^ T-cell functionality and fitness, we thus depleted CD8^+^ T cells in vivo with αCD8 antibody (Supplementary Fig. 3i, j). Notably, tumor volume of WT and Slc7a11-KD tumors remained comparable upon CD8^+^ T-cell depletion (Fig. 3j). In summary, our findings suggest that limiting cystine uptake by tumor cells alleviates T-cell ferroptosis and exhaustion, greatly improving anti-tumor immunity.

### Intratumoral cystine supplementation boosts CD8^+^ T cell anti-tumor immunity

**Fig. 4:**
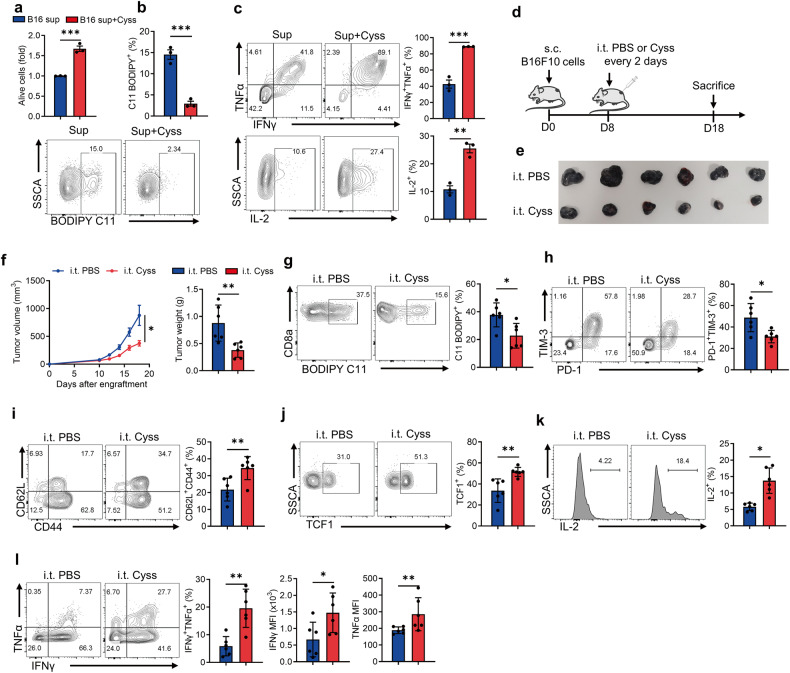
Cystine supplementation inhibits CD8^+^ T-cell ferroptosis and boosts anti-tumor immunity. **a**–**c** CD8^+^ T cells were incubated in B16F10 supernatant with PBS or additional 200 μmol/L cystine for 48 h (*n* = 3 per group), and cell viability was detected by CCK8 assay (**a**). The levels of lipid peroxidation were detected using BODIPY C11 staining (**b**). Cytokine secretion by the indicated T cells was measured by flow cytometry (**c**). **d** Diagram of intratumoral injection of PBS or cystine in B16F10 tumors (*n* = 6 per group). **e**, **f** Tumor size (**e**), tumor volume and tumor weight (**f**) of the indicated groups. **g** The levels of lipid peroxidation in the indicated tumor-infiltrating CD8^+^ T cells. **h**–**j** Flow cytometry analysis of the percentages of PD-1^+^TIM-3^+^ subset (**h**), CD62L^+^CD44^+^ subset (**i**), and TCF1 expression (**j**) in the tumor-infiltrating CD8^+^ T cells. **k**, **l** IL-2 (**k**), IFNγ and TNFα secretion (**l**) of the indicated T cells. Each symbol represents one individual. Data are mean ± s.e.m. *p* values are measured by two-tailed unpaired Student’s *t* test. *p < 0.05, **p < 0.01, ***p < 0.01.

Since CD8^+^ T cells are more sensitive to cystine deprivation compared to tumor cells, we speculated that supplementing cystine in the TME could increase anti-tumor immunity. To this end, we first simulated the TME by culturing CD8^+^ T cells with B16F10 cell supernatant, revealing that cystine supplementation in the supernatant prevented T-cell ferroptosis (Fig. 4a, b) and enhanced T-cell function (Fig. 4c). Next, we sought to investigate the impact of cystine administration on anti-tumor immunity in vivo (Fig. 4d). Indeed, intratumoral cystine supplementation delayed tumor growth (Fig. 4e, f), suppressed T-cell ferroptosis (Fig. 4g) and exhaustion (Fig. 4h). Moreover, cystine addition favored T-cell memory formation and stemness (Fig. 4i, j), and enhanced the cytokine production of tumor-infiltrating CD8^+^ T cells (Fig. 4k, l). Besides, intratumoral cystine administration did not affect splenic CD8^+^ T-cell ferroptosis (Supplementary Fig. 4a), differentiation (Supplementary Fig. 4b–d), and effector function (Supplementary Fig. 4e, f). In summary, intratumoral cystine supplementation prevented T-cell exhaustion and ferroptosis, improving anti-tumor immunity.

### Cystine deprivation triggered CD36-mediated lipid accumulation and CD8^+^ T-cell ferroptosis

Next, we sought to further explore the mechanism underlying T-cell ferroptosis induced by cystine deprivation. The two main triggers of ferroptosis are oxidative stress and lipid accumulation [39]. It is known that cystine is critical for glutathione synthesis to prevent ferroptosis of tumor cells [26]. We confirmed that cystine deprivation impeded T-cell glutathione production (Supplementary Fig. 5a), leading to elevated intracellular ROS and mitochondrial ROS in T cells (Supplementary Fig. 5b, c). Thus, cystine deprivation-induced oxidative stress is the primary factor of T-cell ferroptosis.

**Fig. 5:**
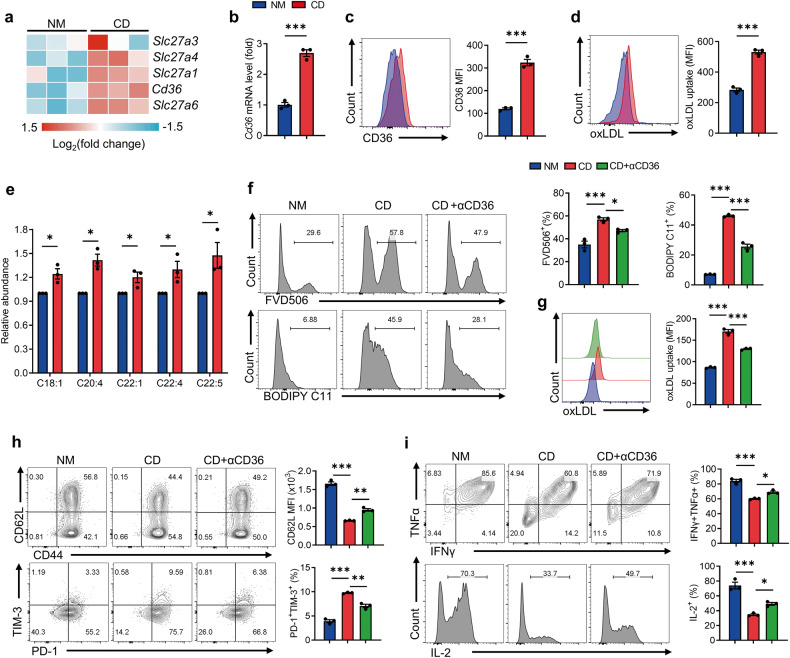
Cystine deprivation upregulates CD36 which causes lipid accumulation and ferroptosis in T cells. **a** RNA-seq analysis of fatty acid uptake-related genes in the indicated T cells (*n* = 3 per group). **b**, **c** RT-qPCR (**b**) and flow cytometry (**c**) analysis of T-cell CD36 expression cultured in NM or CD (*n* = 3 per group). **d** oxLDL uptake assay of the indicated CD8^+^ T cells via oxLDL-DyLight 488 staining (*n* = 3 per group). **e** Polyunsaturated fatty acid levels in the indicated CD8^+^ T cells (*n* = 3 per group). **f** CD8^+^ T cells were cultured in NM, CD, or CD + αCD36 medium for 72 h (*n* = 3 per group). The levels of cell death and lipid peroxidation in the indicated T cells were assessed by flow cytometry. **g**, **h** Flow cytometry analysis of the oxLDL uptake (**g**), the percentages of CD44^+^CD62L^+^ subset, and PD-1^+^TIM-3^+^ subset (**h**) of the indicated T cells. **i** IFNγ, TNFα and IL-2 secretion of the indicated T cells. Each symbol represents one individual. Data are mean ± s.e.m. *p* values are measured by two-tailed unpaired Student’s *t* test (**b**–**e**) and one-way ANOVA with Tukey’s multiple comparison test (**f**–**i**). *p < 0.05, **p < 0.01, ***p < 0.001.

Notably, our data indicated that cystine deprivation disrupted lipid homeostasis in T cells. RNA sequencing analysis showed increased expression of genes related to fatty acid uptake, including CD36, in cystine-deprived T cells (Fig. 5a), while no significant difference was observed in genes associated with fatty acid synthesis or fatty acid oxidation (Supplementary Fig. 5d). CD36, a scavenger receptor orchestrating lipid uptake, including lipids like arachidonic acid (C20:4) and oxidized low-density lipoprotein (oxLDL), has been reported as a key player in triggering T-cell ferroptosis [40, 41]. We confirmed that cystine deprivation in T cells induced CD36 expression at both transcriptional and protein levels (Fig. 5b, c). Consistently, CD36 blockade decreased ferroptosis (Fig. 5f) and reduced oxLDL uptake in T cells during cystine deprivation (Fig. 5g). Furthermore, CD36 blockade partially rescued the Tcm cell subset, reduced the Tex cell subset, and enhanced cytokine generation in cystine-starved T cells (Fig. 5h, i).

In conclusion, cystine deprivation triggers T-cell ferroptosis through diverse mechanisms, inducing both oxidative stress and CD36-mediated lipid accumulation, resulting in T-cell exhaustion and ferroptosis.

### Dysregulated cystine/glutamate exchange upregulates T-cell CD36 expression

**Fig. 6:**
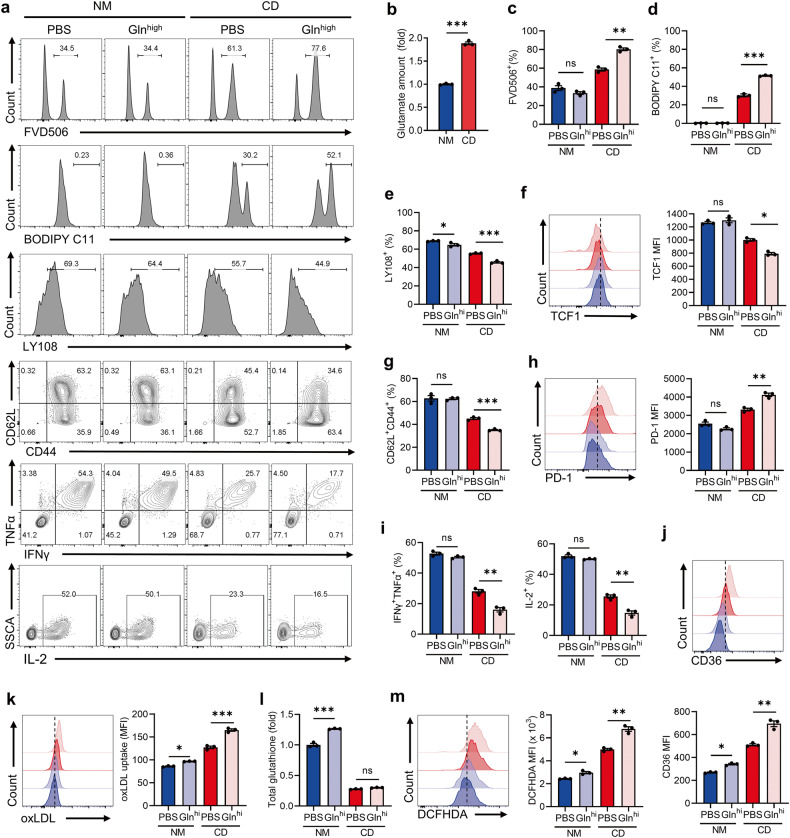
Cystine deprivation triggers glutamate accumulation and CD36 elevation. **a** Representative plots of the FVD506^+^, BODIPY C11^+^, LY108^+^, CD62L^+^CD44^+^ subsets and cytokine-secretion levels of the indicated T cells (*n* = 3 per group). **b** Glutamate content of CD8^+^ T cells in the indicated groups. **c**–**h** T cells were cultured in NM, CD, NM-high glutamine and CD-high glutamine for 48 h. The FVD506^+^ subset (**c**), BODIPY C11^+^ subset (**d**), LY108^+^ subset (**e**), TCF1 expression (**f**), CD62L^+^CD44^+^ subset (**g**), PD-1 expression (**h**), and the levels of cytokine secretion (**i**) in the indicated T cells were measured by flow cytometry. **j** The representative plot (top) and quantitative analysis (bottom) of CD36 expression of the indicated T cells. **k** Flow cytometry analysis of oxLDL-DyLight-488 MFI of indicated T cells. **l** Quantification of total glutathione in the indicated T cells. **m** Intracellular ROS detection of the Indicated T cells incubated with DCFHDA. Each symbol represents one individual. Data are mean ± s.e.m. *p* values are measured by two-tailed unpaired Student’s *t* test (**b**) and one-way ANOVA with Tukey’s multiple comparison test (**c**–**m**). Ns not significant, *p < 0.05, **p < 0.01, ***p < 0.001.

System Xc- mediates the uptake of extracellular cystine in exchange for glutamate [42]. Upon cystine starvation, the compromised function of System Xc- leads to intracellular glutamate accumulation in T cells (Fig. 6b). Previous studies have reported that glutamate accumulation induces ferroptosis in cancer cells [30, 33]. Hence, we aimed to investigate whether glutamate accumulation contributes to T-cell ferroptosis. Intracellular glutamate primarily originates from glutamine via glutaminolysis [43, 44]. Therefore, we cultured T cells in high glutamine concentration (20 mmol/L) to examine the impact of glutamate accumulation on T-cell ferroptosis. Our findings indicate that under cystine starvation, glutamate accumulation exacerbated T-cell ferroptosis (Fig. 6a, c, d), impeded T-cell memory formation and stemness (Fig. 6a, e–g), and increased PD1 expression (Fig. 6h). In addition, high glutamine further aggravates T-cell dysfunction triggered by cystine starvation (Fig. 6a, i). Interestingly, we observed that glutamate accumulation led to CD36 up-regulation in T cells, which could be further elevated in the absence of cystine (Fig. 6j and Supplementary Fig. 6a). Consistent with elevated CD36, oxLDL uptake was increased in T cells under glutamate accumulation (Fig. 6k). RSL3 is a ferroptosis inducer via inhibiting GPX4 activity. We further confirmed that glutamate accumulation-induced CD36 upregulation was also enhanced in response to RSL3 stimulation (Supplementary Fig. 6b). Altogether, these data clarified that glutamate accumulation-induced CD36 upregulation was independent of cystine.

As glutamate is involved in glutathione synthesis, we further examined whether glutamate accumulation facilitates glutathione generation to maintain redox homeostasis. Under cystine deprivation, glutamine supplementation does not support glutathione synthesis in T cells (Fig. 6l). Besides, glutamate serves as a primary substrate for ROS generation [30, 31], and we noticed that glutamate accumulation increased intracellular ROS in cystine-starved T cells (Fig. 6m). Collectively, these results indicated that under cystine starvation, accumulated glutamate instigated CD36 upregulation and elevated oxidative stress, which resulted in T-cell exhaustion and ferroptosis.

### Gclc overexpression protects T cells from ferroptosis and promotes anti-tumor immunity

**Fig. 7:**
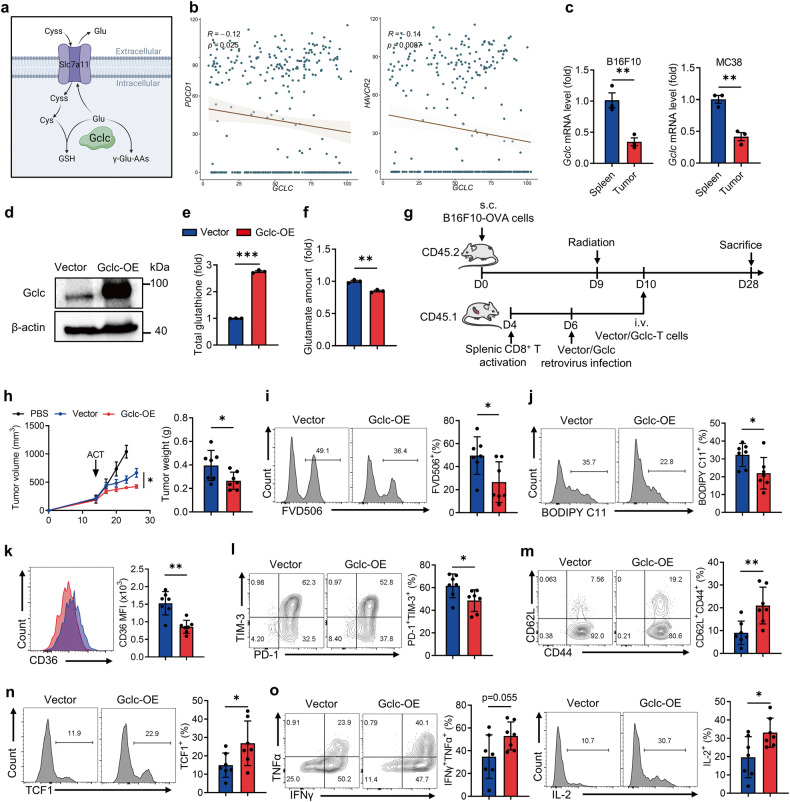
Enforced Gclc expression in CD8^+^ T cells limits ferroptosis and boosts anti-tumor immunity. **a** Diagram of Gclc’s dual role for consuming intracellular glutamate. **b** Correlation analysis of *PDCD1, HAVCR2* and *GCLC* expression in tumor-infiltrating CD8^+^ T cells from melanoma patients (GSE120575 [56]). **c** RT-qPCR analysis of *Gclc* expression of CD8^+^ T cells from B16F10 and MC38 tumors and spleens (*n* = 3 per group). **d** Western blot assay of Gclc expression of the indicated T cells. **e** Quantification of total glutathione in vector and Gclc-OE T cells culturing in NM (*n* = 3 per group). **f** Glutamate content of vector and Gclc-OE T cells cultured in CD (*n* = 3 per group). **g** Diagram of adoptive transfer of vector and Gclc-OE T cells in B16F10-OVA tumors. **h** Tumor volume and tumor weight of the indicated groups (*n* = 7 per group). **i**, **j** The percentages of FVD506^+^ subset (**i**) and BODIPY C11^+^ subset (**j**) of the indicated tumor-infiltrating T cells. **k** CD36 expression of the indicated tumor-infiltrating T cells. **l**–**o** The percentages of PD-1^+^TIM-3^+^ subset (**l**), CD62L^+^CD44^+^ subset (**m**), TCF1^+^ subset (**n**), and the levels of cytokine secretion (**o**) of the indicated tumor-infiltrating T cells. Each symbol represents one individual. Data are mean ± s.e.m. *p* values are measured by two-tailed unpaired Student’s *t* test. *p < 0.05, **p < 0.01.

Based on previous findings, alleviating the glutamate accumulation under cystine starvation shows the potential to counter T-cell exhaustion and ferroptosis. The catalytic subunit of glutamate-cysteine ligase, Gclc, has been reported to ameliorate accumulated glutamate in the absence of cystine, mitigating ferroptosis in tumor cells [33] (Fig. 7a). In this regard, we speculated that Gclc might serve as a critical target to resist T-cell exhaustion and ferroptosis. Notably, *Gclc* expression in CD8^+^ T cells was negatively correlated with the exhaustion marker *PDCD1* and *HAVCR2* in melanoma patients (Fig. 7b). Consistently, *Gclc* expression in mouse tumor-infiltrating CD8^+^ T cells was significantly lower than in splenic CD8^+^ T cells (Fig. 7c).

To elucidate the potential of Gclc in preventing T-cell exhaustion and ferroptosis under cystine deprivation, we generated Gclc overexpression (Gclc-OE) CD8^+^ T cells and their counterparts through retrovirus infection (Fig. 7d and Supplementary Fig. 7a). The infected T cells co-expressed Thy1.1, allowing us to gate on the Thy1.1^+^ subset for the precise examination of vector and Gclc-OE T cells. Under cystine starvation, Gclc-OE T cells exhibited increased glutathione production and glutamate consumption (Fig. 7e, f). Moreover, Gclc-OE T cells cultured in vitro are more resistant to cystine deprivation-induced ferroptosis (Supplementary Fig. 7b, c), accompanied by diminished oxidative stress (Supplementary Fig. 7d), deceased CD36 expression (Supplementary Fig. 7e) and reduced oxLDL uptake (Supplementary Fig. 7f). In addition, Gclc-OE T cells under cystine deprivation exhibited reduced exhaustion phenotype (Supplementary Fig. 7g), improved memory formation (Supplementary Fig. 7h, i), and enhanced cytokine secretion ability (Supplementary Fig. 7j, k).

To assess whether Gclc-OE T cells exhibit improved anti-tumor immunity in vivo, we adoptively transferred CD45.1^+^ Gclc-OE T cells and their counterparts in CD45.2^+^ B16F10-OVA tumor-bearing mice (Fig. 7g). Notably, the adoptive transfer of Gclc-OE T cells significantly suppressed tumor development (Fig. 7h and Supplementary Fig. 7l). Futhermore, Gclc-OE CD8^+^ T cells exhibited reduced ferroptosis at the tumor site (Fig. 7i, j), along with decreased CD36 expression (Fig. 7k). Enforced Gclc expression also prevented T-cell exhaustion (Fig. 7l), promoted memory formation and stemness (Fig. 7m, n), and enhanced T-cell function (Fig. 7o). However, no evident change was observed regarding T-cell ferroptosis (Supplementary Fig. 7m–o), differentiation, and functional state in the DLN (Supplementary Fig. 7p, s).

In conclusion, these findings demonstrate that Gclc facilitates glutathione synthesis and reduces glutamate-induced CD36 upregulation in cystine-starved T cells, thereby shielding T cells from exhaustion and ferroptosis, leading to enhanced anti-tumor immunity.

## Discussion

Elucidating the key factors hindering T-cell immunity in the TME is crucial for developing immunotherapeutic strategies against solid cancers. Our present study highlights the crucial role of sufficient cystine availability in the TME in supporting a productive T-cell immune response. Cystine consumption by tumor cells disrupts cystine/glutamate exchange in CD8^+^ T cells, which thus triggers CD36-mediated lipid accumulation and aberrant ROS production, leading to T-cell exhaustion and ferroptosis. Furthermore, elevated Gclc expression alleviates oxidative stress and glutamate accumulation, significantly enhancing T-cell immunity.

Our study showed that T-cell survival is more sensitive to cystine availability compared to tumor cells. While cystine deprivation in the TME has been reported to inhibit tumor growth by inducing tumor cell ferroptosis [45], our findings suggested that cystine starvation severely impairs T cell anti-tumor immunity. Hence, anti-tumor therapies in terms of cystine depletion should be treated with caution. Notably, different tumor types exhibit varying response to cystine deprivation. For instance, tumor cells with high Nrf2 expression are more resistant to ferroptosis induced by cystine depletion [33]. In such cases, cystine supplementation may enhance immune response and improve therapeutic efficacy. Therefore, future studies are needed to precisely identify the appropriate tumor types for cystine depletion or supplementation therapies. Additionally, our study emphasizes the impact of cystine transport on intracellular lipid accumulation in T cells. CD36 is the key factor inducing T-cell ferroptosis via excessive uptake of lipid peroxides [40, 41]. Our data suggested that cystine/glutamine exchange dysfunction exacerbates CD36-mediated lipid accumulation, consequently inducing T-cell ferroptosis. These findings provide important insights into rational design of future immunotherapy strategies.

The strategies targeting cystine metabolism to enhance T-cell immunity have attracted increasing attention. Several studies have reported that T cells overexpressing either Slc7a11 or cystathionine-gamma-lyase, the key enzyme converting methionine to cysteine, exhibit enhanced tumor suppression [17, 46]. Our study presents a strategy for T cells to counteract cystine deprivation. Gclc overexpression not only counters oxidative stress induced by cystine deficiency, but also mitigates the damage caused by cystine/glutamine exchange dysfunction, thereby enhancing T cell anti-tumor effects. This was in line with previous studies that heightened Gclc expression improves redox balance and prolongs mammalian cell lifespans [47–50]. Conversely, Gclc ablation in T cells severely impairs inflammatory responses [21]. Furthermore, overexpression of Nrf2, a key regulator of Gclc, significantly enhances T cell anti-tumor functionality [51]. Collectively, these findings highlight Gclc as a potential therapeutic target for boosting T cell anti-tumor immunity.

Nevertheless, this study has certain limitations. While the decrease of cystine in the TME has been demonstrated, the specific concentration of cystine may vary across different tumor types [17, 23]. Besides, plasma amino acid levels undergo dynamic changes as the tumor progress [52], implying that amino acid abundance in the TME may be also influenced by tumor development. Thus, the in vitro setting used in this study to investigate the impacts of cystine deprivation on T cells may not fully capture the dynamic and intricate TME. The variations in cystine content across different tumor types or stages should also be taken into account in future studies. Furthermore, while analysis from specific human cancer datasets supports our findings, the impact of cystine deprivation on T cells in human cancers requires further investigations.

In conclusion, our results reveal the impaired T cell-immunity due to cystine competition by tumor cells. We elucidate a novel mechanism of ferroptosis in tumor-infiltrating T cells: cystine deprivation leads to glutamate accumulation, subsequently exacerbating CD36-mediated lipid peroxides production. These findings provide a theoretical foundation for immunotherapeutic strategies targeting amino acid metabolism or CD36 blockade. Furthermore, we prove that elevated Gclc expression protects T cells from cystine deprivation induced damage, presenting a potential strategy for engineering T cells for effective anti-tumor immunotherapy.

## Materials and methods

### Cell lines

HEK-293FT cells (#CRL-3249, ATCC, USA) and MC38 cells (#ENH204-FP, Kerafast, USA) were cultured in DMEM (#C11995500CP, Gibco, USA) containing 10% FBS (#SH30396, Cytia, USA), and penicillin-streptomycin (#SV30010, Hyclone, USA) at 37 °C with 5% CO_2_. B16F10-OVA cells were kindly provided by Professor Bo Huang (Institute of Basic Medical Sciences, Chinese Academy of Medical Sciences and Peking Union Medical College, Beijing, China). B16F10 cells (#CRL-6475, ATCC, USA) and B16F10-OVA cells were cultured in RPMI (#11875-093, Gibco, USA) containing 10% FBS and penicillin-streptomycin at 37 °C with 5% CO_2_. Cell lines were regularly checked for mycoplasma contamination.

### Mice

The animal protocol was approved by the Institutional Animal Care and Use Committee (IACUC) of Suzhou Institute of Systems Medicine (ISM-IACUC-0018 and ISM-IACUC-0055). We confirm that all experiments were conducted in accordance with the relevant regulations of the committee. CD45.1^+^ OT-1 TCR transgenic mice (C57BL/6 N background) and CD45.2^+^ female C57BL/6 N mice (6–8 weeks old, WT) were purchased from Vital River Company (Beijing, China) and housed under specific pathogen-free conditions at Suzhou Institute of Systems Medicine.

### Quantification of cystine concentration

Tumor interstitial fluid was isolated from B16F10 tumors as previously described [23]. Serum was obtained by centrifuging blood at 3500 rpm for 5 min and collecting the supernatant. Cystine concentration was quantified by liquid chromatography-tandem mass spectrometry (LC-MS). LC-MS analysis was conducted at the APTBIO Biotechnology Inc (Shanghai, China).

### CD8^+^ T-cell isolation and activation

OT-1 mouse spleens were processed into single-cell suspensions through 40 μm filters. The splenocytes were incubated in 2 mL of red blood cell lysis buffer (#420301, Biolegend, USA) for 10 min. Subsequently, splenocytes were washed by PBS and resuspended at 1 × 10^6^/mL in T-cell medium (formula listed below) containing OVA peptide (1 μg/mL, #SP1050a, Abcepta Biotech, USA) and IL-2 (10 ng/mL, #200-02-500, Pepro Tech, USA) to activate CD8^+^ T cells. After 3 days, dead cells were removed by Ficoll-Paque separation (#GE17-1440-03, Sigma-Aldrich, USA), and alive CD8^+^ T cells were collected for subsequent experiments.

Formula of T-cell medium: The RPMI medium contained 10% FBS (#SH30396, Cytiva, USA), penicillin-streptomycin (1:100, #SH40003, Cytiva, USA), Hepes (10 mmol/L, #03-025-1B, Biological Industries, Israel), Sodium pyruvate (1 mmol/L, #11360-070, Gibco, USA), MEM non-essential amino acids (1:100, #11140-050, Gibco, USA), L-glutamine (2 mmol/L, #25030081, Gibco, USA), and β-mercaptoethanol (5 μmol/L, #B6891, Sigma, USA).

### Cystine deprivation in CD8^+^ T cells

The RPMI medium lacking cystine, methionine, and glutamine was customized from Duoning Biological Company (Shanghai, China). Methionine (100 μmol/L, #M9625, Sigma, USA), cystine hydrochloride (#C6727, Sigma, USA), and other reagents according to the T-cell medium formula were added to the customized medium. Activated T cells were cultured in either normal (200 μmol/L) or cystine-deprived (20 μmol/L) medium with IL-2 and IL-7 (10 ng/mL) for 72 h.

### Sensitivity of T cells and tumor cells to cystine deprivation

Activated T cells, B16F10 cells, and MC38 cells were cultured separately in T-cell medium with varying concentrations of cystine (0.001, 0.01, 0.1, 1, 10, 20, 40, 70, 100, 150, and 200 μmol/L). After 48 h of incubation, the CCK8 reagent (1:100, #K1018, APE-BIO, USA) was added to measure the cell viability.

### T-cell cultivation in tumor supernatant

B16F10 and MC38 cells were cultured for 48 h in T-cell medium with cystine concentrations of 200, 50, and 30 μmol/L, and the medium (tumor supernatant) was collected. Subsequently, activated T cells were cultured for 24 h in either fresh medium or tumor supernatants with 200, 50, and 30 μmol/L cystine. The percentage of dead cells was assessed using flow cytometry.

### Tumor and T cell co-culture

B16F10 and MC38 tumor cells were planted in 24-well plates (10^6^ cells per well). After cell adhesion, the medium was replaced with either normal medium or 20 μmol/L cystine-deprived medium. Subsequently, 10^6^ activated T cells were added to the upper chamber of transwell inserts, which were then placed into the 24-well plate for co-culture with tumor cells (Supplementary Fig. 1g). After 24 h, T cells from the upper chambers were collected, and cell viability was assessed using the CCK-8 assay.

### Cell death pathway inhibition

Activated T cells were individually cultured with 10 μmol/L cystine and the following regents: ferrostatin-1 (10 μmol/L, #HY-100579, MCE, USA), N-Acetyl Cysteine (10 mmol/L, HY-B0215, MCE, USA), Z-VAD-FMK (10 μmol/L, #HY-16658B, MCE, USA), necrostatin-1 (10 μmol/L, #HY-15760, MCE, USA), MCC950 (10 μmol/L, #HY-12815, MCE, USA). Additionally, T cells were cultured in normal medium as control. After 48 h, T-cell viability was detected by CCK-8 and lipid peroxidation was detected by flow cytometry assay.

### CD36 blockade of T cells

Activated T cells were cultured in normal medium, cystine-deprived medium (20 μmol/L), and cystine-deprived medium supplemented with CD36 antibody (10 μg/mL, #163002, Biolegend, USA) for 48 h. Subsequently, T cells were collected for flow cytometry analysis.

### Glutamine supplementation and RSL3 treatment

For glutamine supplementation treatment, activated T cells were cultured in normal medium, normal medium with 20 mmol/L glutamine (#25030081, Gibco, USA), cystine-deprived medium, and cystine-deprived medium with 20 mmol/L glutamine for 48 h. For RSL3 treatment, activated T cells were cultured in normal medium, normal medium with 20 mmol/L glutamine, normal medium containing RSL3 (10 nmol/L, #HY-100218A, MCE, USA), and normal medium containing RSL3 and 20 mmol/L glutamine for 48 h.

### Intratumoral cystine supplementation assay

C57BL/6 N mice were subcutaneously inoculated with 5 × 10^5^ B16F10 cells, because B16F10 tumor was loose and soft, allowing effective intratumoral injection. On day 10 after tumor inoculation, 0.1 mg cystine hydrochloride in 100 μL PBS or PBS alone was intratumoral administered to each mouse every other day and the tumor size was measured. Tumor volume was calculated using the formula V = (L × W^2^) / 2, V is tumor volume, L is tumor length, and W is tumor width. Tumor size did not exceed 2 cm in any dimension in any of the experiments.

### WT and Slc7a11-KD tumor inoculation

To generate pLKO.1-Slc7a11shRNA plasmid, forward oligonucleotide (5′-CCGGGCCCTGTCCTATGCAGAATTACTCGAGTAATTCTGCATAGGACAGGGCTTTTTG-3′) was annealed with reverse oligonucleotide (5′-AATTCAAAAAGCCCTGTCCTATGCAGAATTACTCGAGTAATTCTGCATAGGACAGGGC-3′), and then subcloned into an AgeI (#R0552S, NEB, USA)/EcoRI (#R0101S, NEB, USA) digested pLKO.1-TRC cloning vector (#10879, Addgene, USA). To produce pLKO.1 Lentiviral particles, 293FT cells were co-transfected in a 4:3:1 ratio with pLKO.1-shRNA (or pLKO.1-Slc7a11shRNA), psPAX2, and pMD2.G. The supernatant containing the viral particles was collected 48 and 72 h post-transfection. B16F10 cells were infected with the virus supernatant for 48 h, followed by an additional 7-day selection with 500 μg/mL G418 (#10131035, Invitrogen, USA). Subsequently, C57BL/6 N mice were randomly inoculated with either 5 × 10^5^ WT or Slc7a11-KD B16F10 cells, and tumor volumes were measured since the 10th day post-inoculation. For CD8 depletion assay, 200 μg CD8 antibody was intraperitoneally injected into each mouse three days before tumor inoculation and on days 1, 7, and 12 post-inoculation (Supplementary Fig. 3j).

### Gclc-overexpressing T cell generation and adoptive T-cell therapy

The Gclc sequence was amplificated (Forward primer: 5′-GGGTGGACCATCCTCTAGCCCTCGAGATGGGGCTGCTGTCCCAAG-3′; Reverse primer: 5′-GCTTCCGGCTAGCCCTGCGCAAGCTTCCGGCTGAAGGGTCGCTTTTACCTC-3′) and inserted into XhoI (#R0146L, NEB, USA) and HindIII (#R3104S, NEB, USA) digested pMSGV-Thy1.1 plasmid. pCL-Eco (#HG-VNC0832, Clontech, USA) and pMSGV-Thy1.1 were 1:1 co-transfected into 293FT cells. The virus-containting supernatant was collected at 48 and 72 h post-transfection. Naïve CD8^+^ T cells were sorted from mouse spleens using the CD8^+^ naïve T-cell enrichment kit (#480044, Biolegend, USA), and were planted into αCD3 (1 μg/mL, #16-0031-86, Invitrogen, USA) and αCD28(1 μg/mL, #16-0281-85, Invitrogen, USA) precoated plate. On the 3rd day, activated T cells were planted into RetroNectin (0.1 mg/mL, #T100A, TaKaRa, Japan) precoated 24-well plates, culturing with a 1:1 mixture of virus supernatant and T-cell medium containing polybrene (1 μg/mL, #SC-134220, Santa Cruz, USA) and IL-2 (10 ng/mL). After 24 h, the infected T cells were cultured in T-cell medium. After 48 h, the frequency of Thy1.1^+^ T cells was detected as infection efficiency.

For adoptive T-cell therapy, 5 × 10^5^ B16-OVA tumor cells were subcutaneously injected into CD45.2^+^ C57BL/6 mice. Mice were 5 Gy irradiated when the tumor diameter was approximately 4–5 mm. Subsequently, vector or Gclc-OE T cells were intravenously transferred into recipients (1 × 10^6^ cells per mouse). Tumor size was measured every 3 days.

### Flow cytometry analysis

Spleens, lymph nodes, and tumor tissues were processed into single-cell suspensions using 40μm filters. Lymphocytes from tumor tissues were enriched using Percoll density gradient media (#17089109, Cytiva, Sweden). All samples were stained with Fixable Viability Dye eFluor™ 506 (#65-0866-18, Invitrogen, USA) on ice for 20 min. For cell surface staining, antibodies (listed in Supplementary Table 1) were diluted to 1:200 in FACS buffer (PBS with 2% FBS) and incubated on ice for 25 min. For intracellular cytokine staining, cells were incubated with Brefeldin A (1:1000, #00-4506-51, Invitrogen, USA), Monensin (1:1000, #00-4505-51, Invitrogen, USA), PMA (10 ng/mL, #P8139, Sigma, USA) and ionomycin (500 ng/mL, #FMS-FZ208, Fcmacs, Nanjing, China) at 37 °C for 3.5 h. The cells were then fixed on ice with Fixation Buffer (#420801, Biolegend, USA) for 20 min, and stained with cytokine antibodies (listed in Supplementary Table 1) in permeabilization buffer (#421002, Biolegend, USA). For intracellular transcription factor staining, cells were fixed with Foxp3/Transcription Factor Staining Buffer (#00-5223-56, #00-5123-43, Invitrogen, USA), and then stained with transcription factor antibodies (listed in Supplementary Table 1) in Foxp3/Transcription Factor Permeabilization Buffer (#00-8333-56, Invitrogen, USA). All samples were resuspended in FACS buffer, loaded in an LSR Fortessa flow cytometer (Becton-Dickinson, San Jose, CA) and analyzed using FlowJo software.

### oxLDL uptake and lipid peroxidation assay

To measure oxLDL uptake, cells were incubated at 37 °C in PBS containing oxLDL-DyLight-488 (1:1000, #601181, Cayman, USA) for 20 min. To detect lipid peroxidation, cells were incubated at 37 °C in PBS containing BODIPY FLC11 (1.5 μmol/L, #D3861, Invitrogen, USA) for 20 min. After incubation, the cells were washed with FACS buffer for cell surface staining.

### ROS and glutathione measurement

For ROS measurement, T cells were incubated at 37 °C for 20 min in PBS containing DCFHDA (1:1000, #S0033S, Beyotime, China) for cellular ROS detection or with Mito-SOX (1:200, #M36008, Invitrogen, USA) for mitochondrial ROS detection. After incubation, T cells were resuspended in FACS buffer for cell surface staining. For glutathione measurement, 10^6^ alive T cells were collected for each group, and the total glutathione levels were determined according to the protocol of the GSH and GSSG assay kit (#S0053, Beyotime, China).

### RNA sequencing

Total RNA from CD8^+^ T cells were extracted using the RNeasy Mini Kit (#74104, Qiagen, Germany). Three biological replicates were performed for each studied condition. RNA sequencing was conducted and the data were processed by the bioinformatic core at the Suzhou Institute of Systems Medicine. Source data were published on the Figshare website, Doi: 10.6084 / m9 Figshare. 24925728. Gene sets related to lipid peroxidation/ferroptosis activation, T-cell memory/effector differentiation, and T-cell dysfunction/exhaustion were selected from existing publications [53–55]. Heatmap analysis were performed by Hiplot Pro (https://hiplot.com.cn/), a comprehensive web service for biomedical data analysis and visualization.

### Quantitative lipidomics assay

T cells were cultured in NM or CD for 3 days, and the alive cells were collected for lipidomics assay. LC-MS analysis was conducted at the Metware Biotechnology Inc (Wuhan, China). For the detection of free fatty acids, the samples were homogenized and mixed with an internal standard mixture in ice-cold methanol. The extracted free fatty acids were then converted to acyl chloride intermediates and detected using previously described methods [40].

### Real-time quantitative PCR and western blot assay

CD8^+^ T cells from spleens or tumor tissues were sorted using PE-anti-CD45.1 antibody (#110708, Biolegend, USA) and anti-PE magnetic beads (#480080, Biolegend, USA), or T cells from in vitro culture were collected for RT-qPCR. Total RNA was isolated using the RNeasy Mini Kit, and subjected to reverse transcription with PrimeScript RT Master Mix Kit (#RR036B, TaKaRa, Japan). Reactions were conducted on the LightCycler 480 system (Roche, Switzerland) using Perfectstart Green qP CR SuperMix (#AQ601-04, TransGen Bitotech, China). Specific primers are listed in Supplementary Table 2.

For western blot assay, cells were lysed using RIPA buffer (#P0013B, Beyotime, China). Protein was quantified by BCA protein quantification kit (#P0010S, Beyotime, China) and denatured at 95 °C for 10 min. Protein was separated via 10% SDS-polyacrylamide gel and then transferred to PVDF membranes. The membranes were blocked in 5% non-fat milk for 1 h and then incubated with specified primary antibodies (listed in Supplementary Table 1) at 4 °C overnight. After incubation with HRP-secondary antibody, target protein was visualized by the chemiluminescent detection kit (#180–501, Tanon, China) and a ChemiDoc western imaging system (Bio-rad, USA).

### Statistical analysis

All data were analyzed from at least three experiments and presented as mean ± s.e.m. The sample size was not predetermined using a statistical method, it was based on prior experimental observations. Mice were randomly allocated to experimental groups and no blinding method was used for animal experiments. Differences were assessed by two-tailed student’s unpaired *t*-test among two groups. One-way ANOVA with Turkey’s multiple comparison test was used to examine difference between more than two groups. The variance is similar between the groups undergoing statistical comparisons. *p* value < 0.05 was considered significant (ns, no significant, *p < 0.05; **p < 0.01; ***p < 0.001). All statistics were performed using GraphPad Prism 9 (GraphPad, San Diego, USA).

### Supplementary information

Supplemental Figures and Tables

Supplementary File Uncropped WB

Reproducibility Checklist

## Data Availability

Data reported in this paper will be shared by the lead contact upon request. The analysis of the correlation between *Slc7a11* and immune infiltration in human tumors was performed by the TIMER2.0 online database (http://timer.cistrome.org/). The scRNA sequencing data from melanoma patients were found at the GEO accession number GSE120575.
