# Supplementary material for: Cystine deprivation triggers CD36-mediated ferroptosis and dysfunction of tumor infiltrating CD8+ T cells
Source: Cell Death Dis. 2024 Feb 15;15(2):145. doi: 10.1038/s41419-024-06503-1 (PMC10869360; doi:10.1038/s41419-024-06503-1)
Supplement: Supplementary file 1 — Supplemental Figures and Tables [file 41419_2024_6503_MOESM1_ESM.docx]

**Supplementary Information for**

**Cystine Deprivation Triggers CD36-Mediated Ferroptosis and Dysfunction of Tumor Infiltrating CD8^+^ T cells**

Chenfeng Han^#^, Minmin Ge^#^, Pengfei Xing^#^, Tian Xia, Cangang Zhang, Kaili Ma, Yifu Ma, Shicheng Li, Wenhui Li, Xiaowei Liu, Baojun Zhang*, Liyuan Zhang*, and Lianjun Zhang*

This file includes:

Supplementary Fig.1. Cystine deprivation impairs CD8^+^ T-cell survival and cytokine production.

Supplementary Fig.2. Cystine deprivation induces T-cell ferroptosis.

Supplementary Fig.3. Inhibition of cystine uptake by tumor cells affects CD8^+^ T cells.

Supplementary Fig.4. Cystine supplementation does not affect splenic T-cell differentiation and ferroptosis.

Supplementary Fig.5. Lack of cystine reduces glutathione and exacerbates oxidative stress in CD8^+^ T cells.

Supplementary Fig.6. Cystine deprivation induces CD36 upregulation in CD8^+^ T cells.

Supplementary Fig.7. Gclc-OE T cells prevent cystine deprivation induced ferroptosis.

Supplementary table 1. Antibodies used for flow cytometry and WB analysis.

Supplementary table 2. Forward and reverse primers for RT-qPCR.


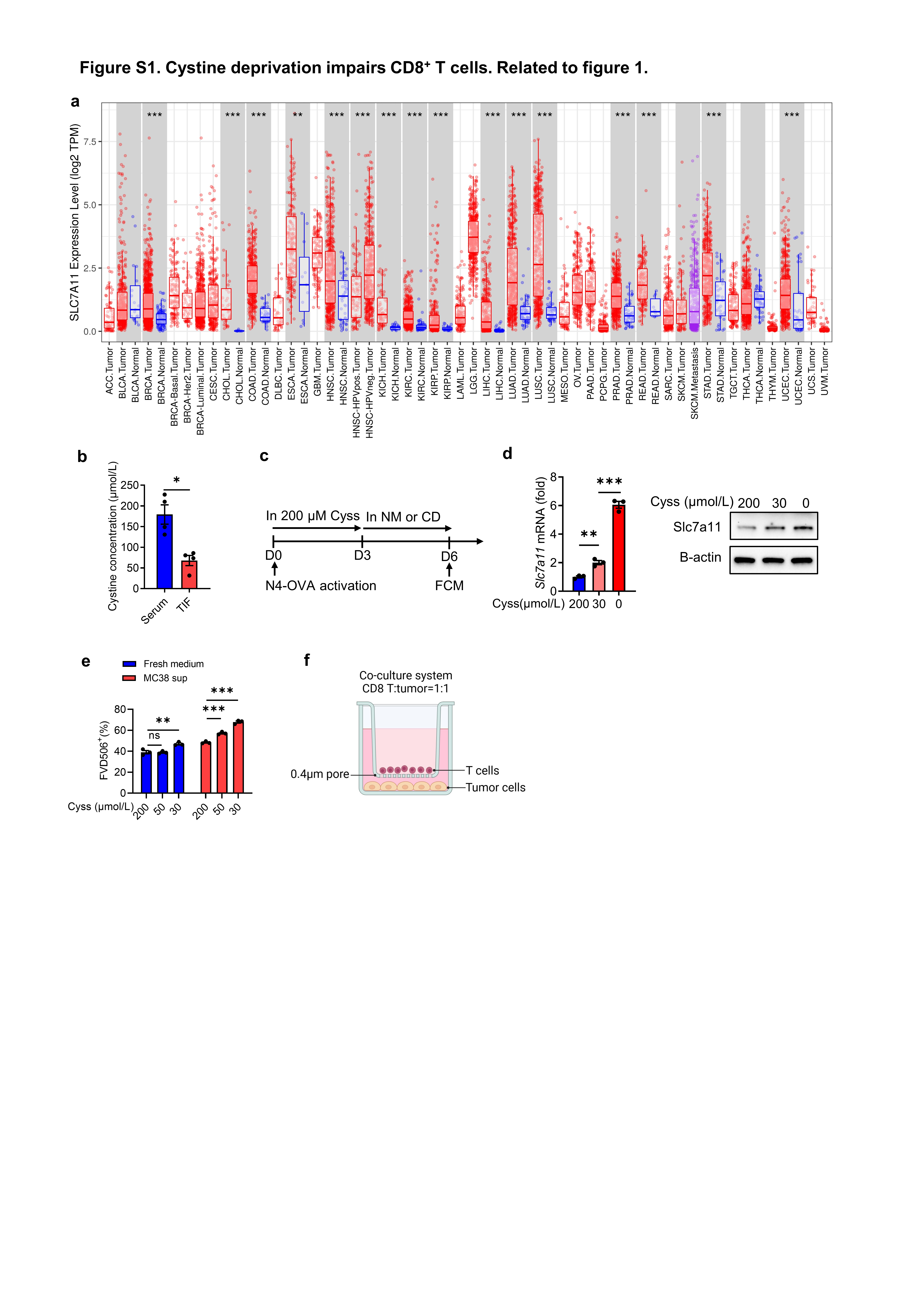


**Supplementary figure 1. Cystine deprivation impairs CD8^+^ T cell survival and cytokine production. Related to figure 1.**

**a,** Analysis of *SLC7A11* expression in multiple tumor and normal tissues using TIMER2.0 database. **b,** The cystine concentration in mouse serum and B16F10 tumor interstitial fluid (TIF). **c,** Diagram of T cell activation and culture. CD8^+^ naive T cells were activated by N4-OVA peptide in NM for 3 days, and then were cultured with NM or CD for another 3 days. **d,** Slc7a11 expression of T cells cultured with varying concentrations of cystine. **e,** CD8^+^ T cells were cultured in fresh medium or MC38 supernatant for 24h with varying concentrations of cystine, and the percentages of FVD506^+^ T cells were detected by flow cytometry. **f,** Diagram illustrating the co-culture of tumor cells and T cells in the Transwell system. Each symbol represents one individual, n=3 per group. Data are mean ± s.e.m.. *p* values are measured by two-tailed unpaired Student’s *t* test (b) and one-way ANOVA with Tukey’s multiple comparison test (d, e). ns, not significant, *p<0.05, ***p*<0.01, ****p*<0.001.


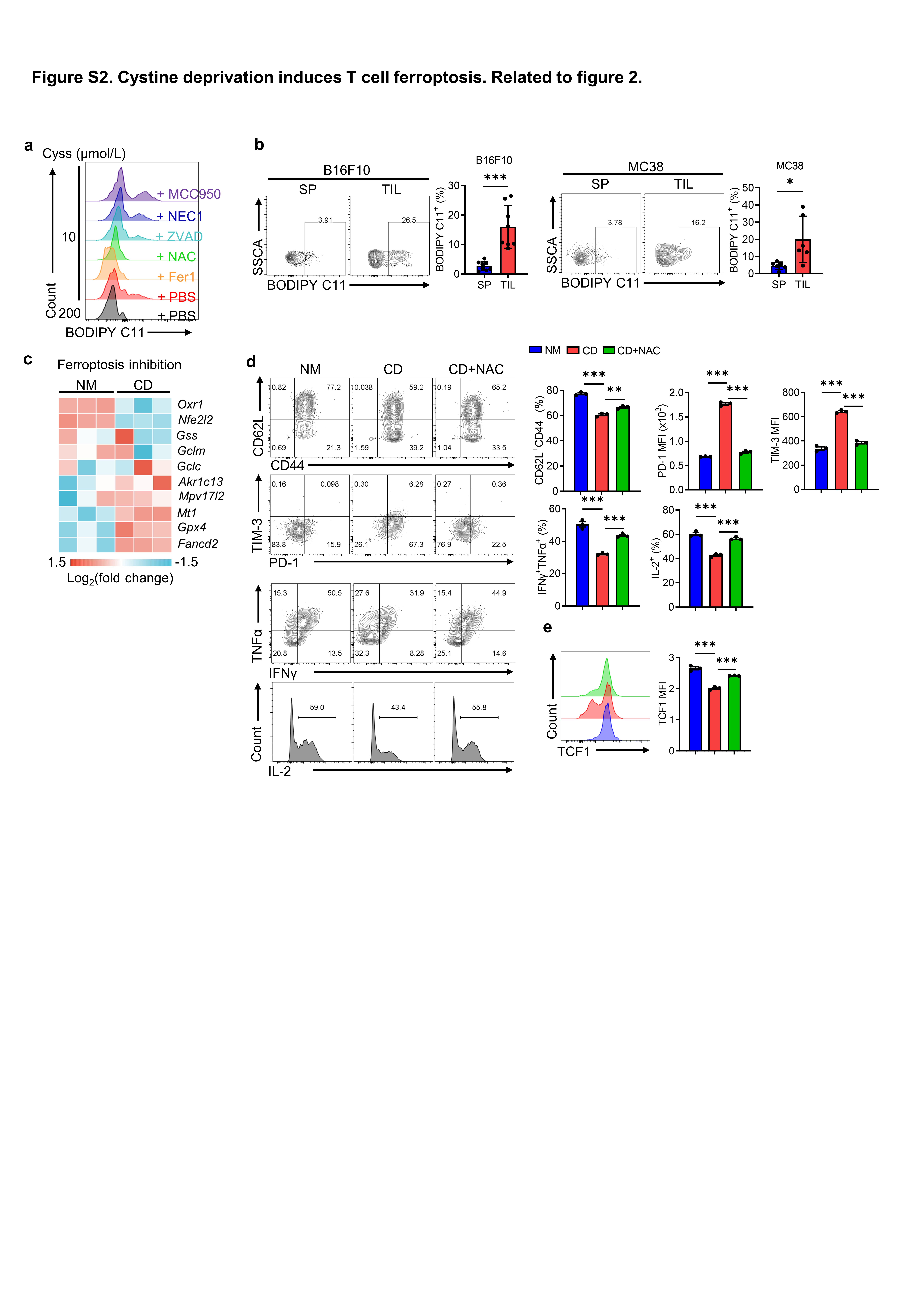


**Supplementary figure 2. Cystine deprivation induces T cell ferroptosis. Related to figure 2.**

**a,** Flow cytometry plots of the effects of cell death inhibitors on lipid peroxidation levels in the indicated T cells (n=3 per group). **b,** Lipid peroxidation levels of splenic CD8^+^ T cells and CD8^+^ tumor-infiltrating T lymphocytes (TIL) in B16F10 (left, n=8 per group) and MC38 (right, n=6 per group) tumors. **c,** RNA-seq analysis of ferroptosis inhibition-related genes of the indicated T cells. **d, e,** T cells cultured in NM, CD, and CD+NAC for 48 hours, and the percentages of CD62L^+^CD44^+^ subset, the levels of PD-1 and TIM-3 expression, cytokine secretion (d) and the TCF1 expression (e) were measured (n=3 per group). Each symbol represents one individual. Data are mean ± s.e.m.. *p* values are measured by two-tailed unpaired Student’s *t* test (b) and one-way ANOVA with Tukey’s multiple comparison test (d, e). **p*<0.05, ***p*<0.01, ****p*<0.001.


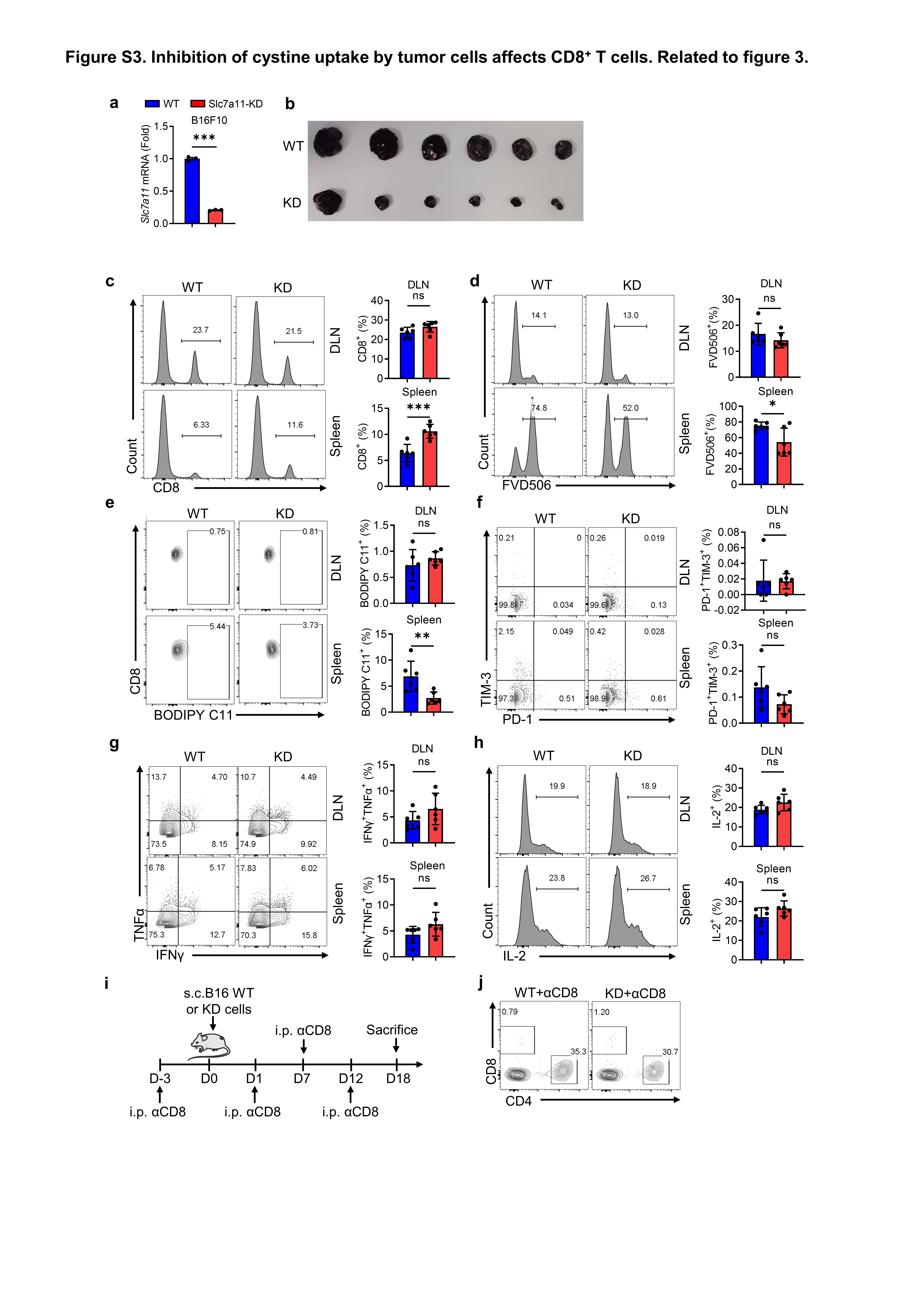


**Supplementary figure 3.** **Inhibition of cystine uptake by tumor cells affects CD8^+^ T cells. Related to figure 3.**

**a,** RT-qPCR analysis of *Slc7a11* expression in WT and Slc7a11-KD B16F10 tumor cells (n=3 per group). **b,** Images of the B16F10-WT and KD tumor sizes. **c-h,** Effects of Slc7a11-KD of tumor cells on CD8^+^ T cells in spleens and draining lymph nodes (DLN). The CD8^+^ T cell proportion (c), dead T-cell subset (d), lipid peroxidation levels (e), PD-1^+^TIM-3^+^ subset (f), and the levels of IFNγ, TNFα (g) and IL-2 secretion (h) of T cells in DLN and spleens were measured by flow cytometry (n=6 per group). **i,** Diagram of intraperitoneal injection of CD8 antibody. (WT+αCD8: n=5; KD+αCD8: n=6). **j,** Representative plots of the CD8^+^ T-cell clearance after CD8 antibody administration. Each symbol represents one individual. Data are mean ± s.e.m.. *p* values are measured by two-tailed unpaired Student’s *t* test. ns, not significant, **p*<0.05, ***p*<0.01, ****p*<0.001.


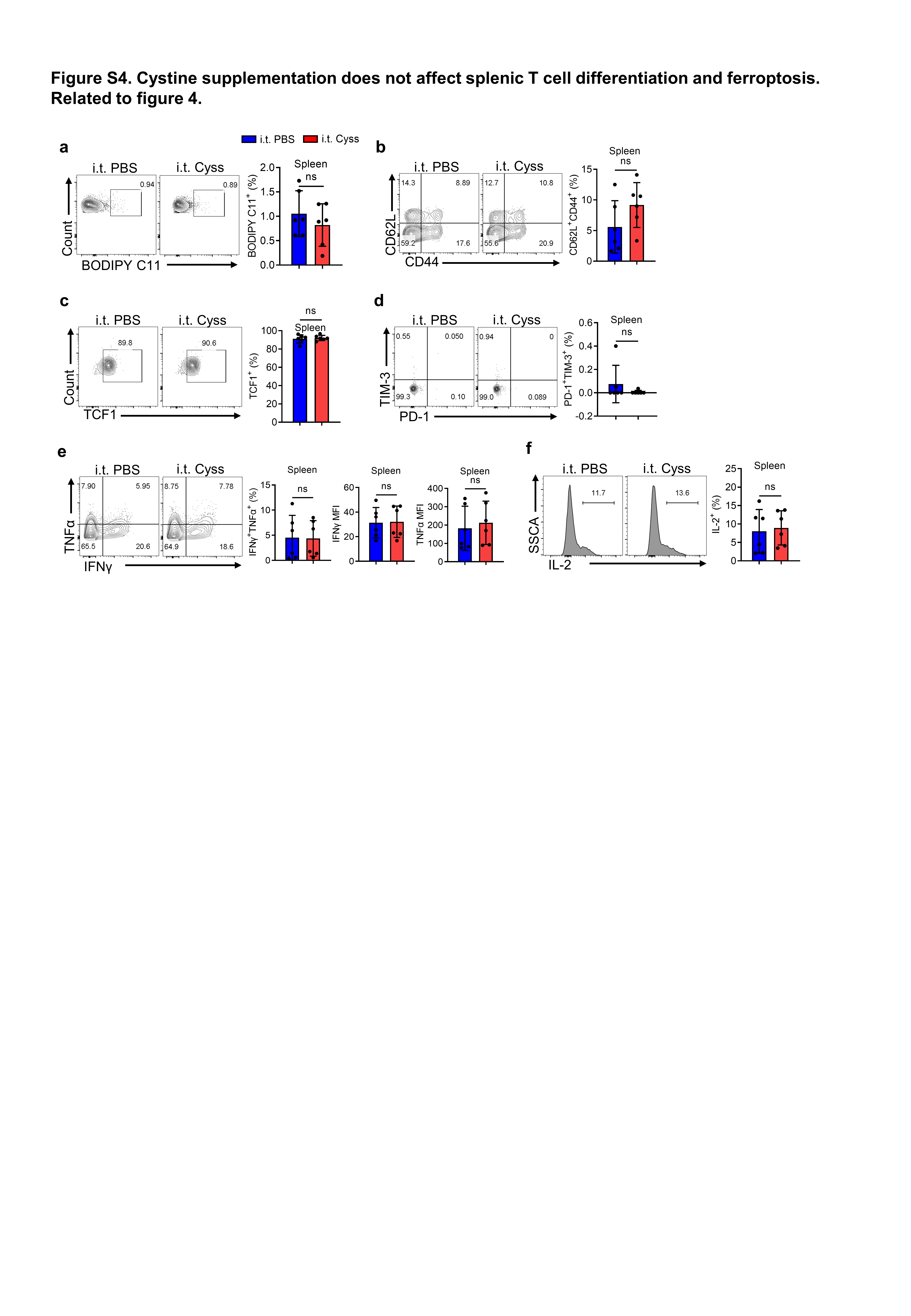


**Supplementary figure 4. Cystine supplementation does not affect splenic T-cell differentiation and ferroptosis. Related to figure 4.**

**a-f,** Splenic T cells corresponding to the B16F10 tumors supplemented PBS or cystine were collected for flow cytometry. Lipid peroxidation of the indicated splenic CD8^+^ T cells were detected by BODIPY C11 staining (a). The percentages of CD62L^+^CD44^+^ subset (b), TCF1 expression (c), and the percentage of PD-1^+^TIM-3^+^ subset (d), and IFNγ, TNFα (e) and IL-2 (f) secretion of the indicated splenic CD8^+^ T cells were detected by flow cytometry. Each symbol represents one individual, n=6 per group. Data are mean ± s.e.m.. *p* values are measured by two-tailed unpaired Student’s *t* test, ns, not significant.


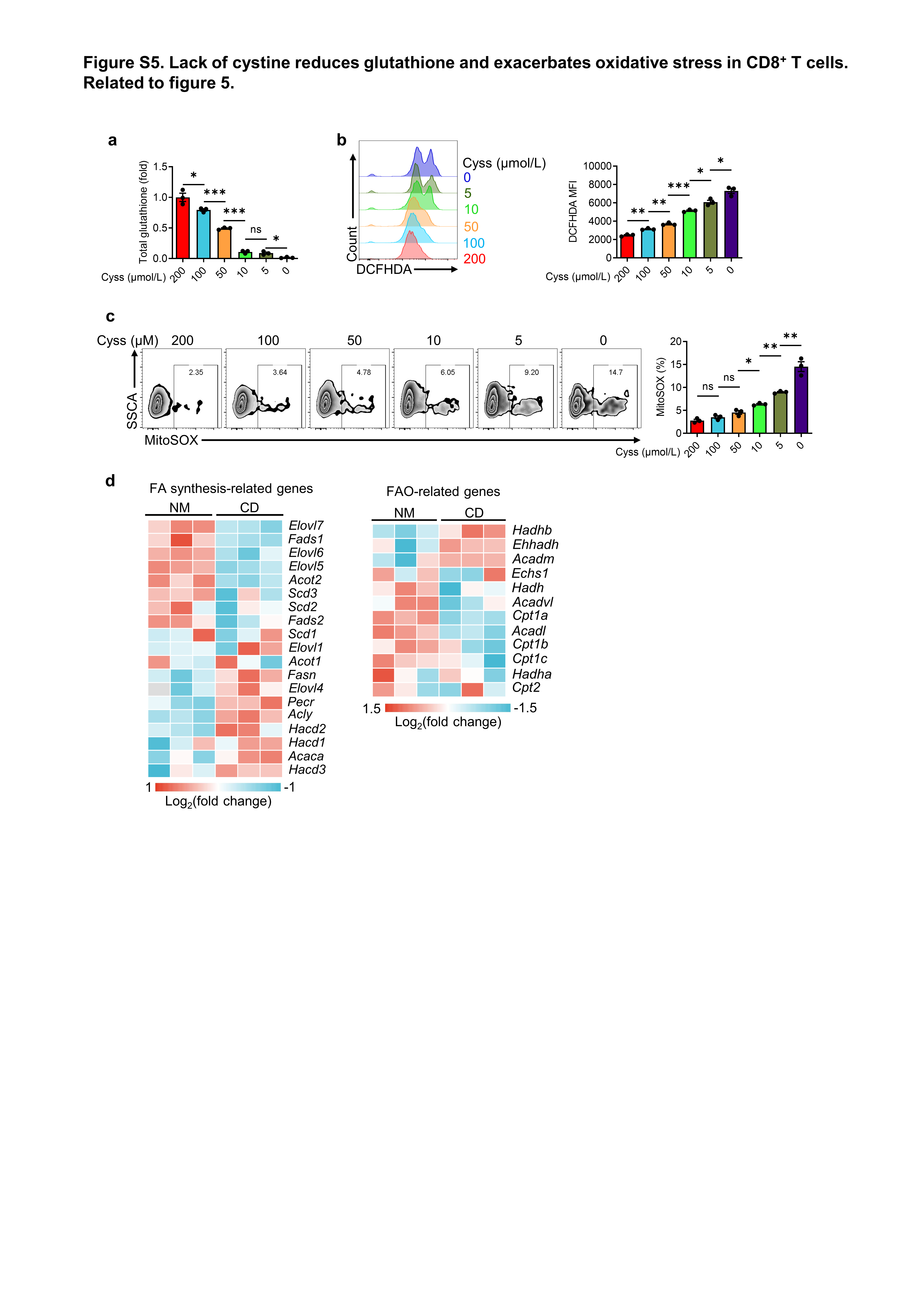


**Supplementary figure 5. Lack of cystine reduces glutathione and exacerbates oxidative stress in CD8^+^ T cells. Related to figure 5.**

**a,** Total glutathione levels of T cells cultured with varying cystine concentrations. **b,** Flow cytometry analysis of intracellular ROS levels in the indicated T cells via DCFHDA staining. **c,** Flow cytometry analysis of mitochondrial ROS in the indicated T cells via MitoSOX staining. **d,** RNA-seq analysis of fatty acid synthesis-related genes (left) and fatty acid oxidation (FAO)-related genes (right) of the indicated T cells. Each symbol represents one individual，n=3 per group. Data are mean ± s.e.m. . *p* values are measured by one-way ANOVA with Tukey’s multiple comparison test, ns, not significant. **p*<0.05, ***p*<0.01, ****p*<0.001.


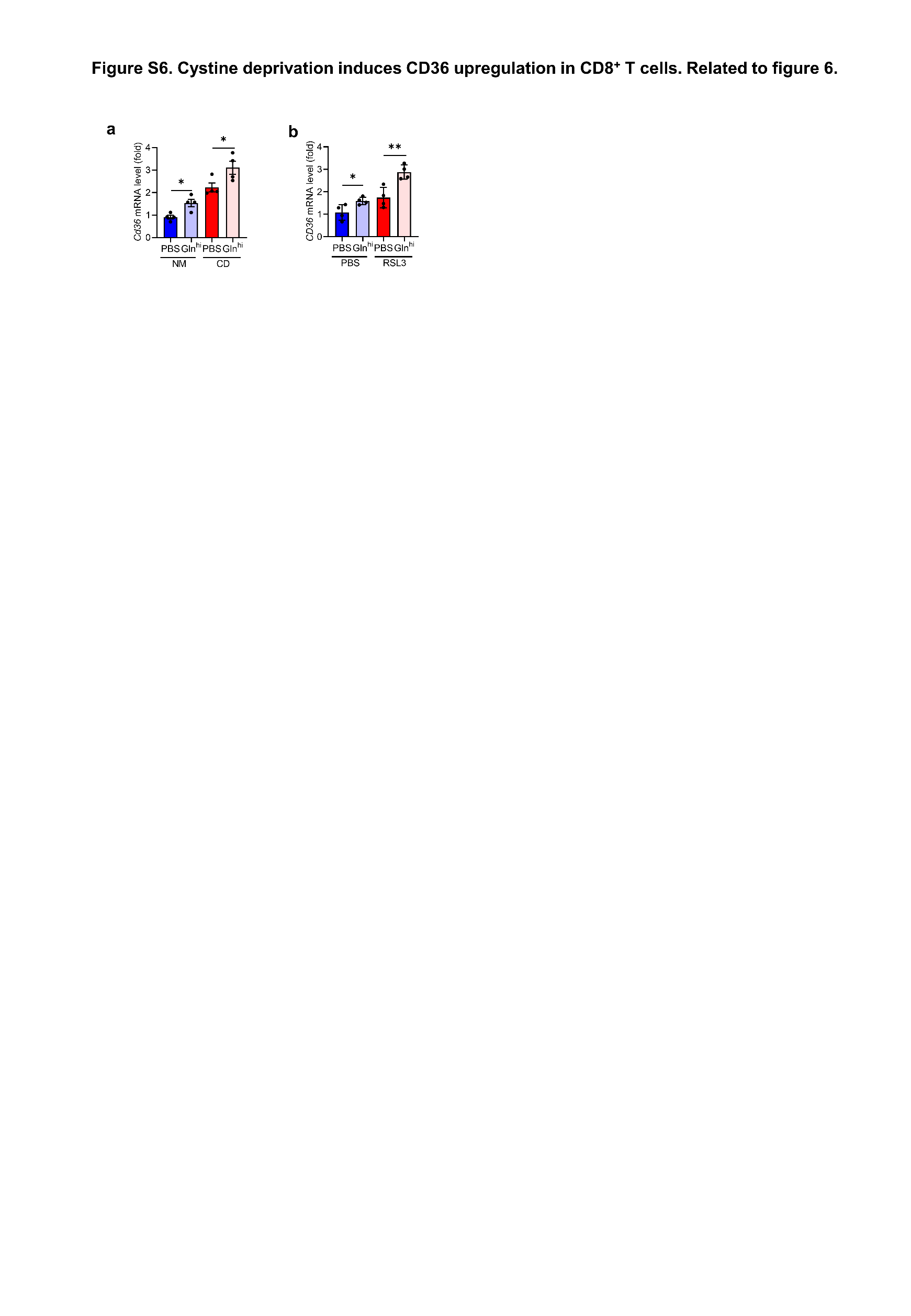


**Supplementary figure 6. Cystine deprivation induces CD36 upregulation in CD8^+^ T cells. Related to figure 6.**

**a,** RT-qPCR analysis of CD36 expression in NM or CD treated CD8^+^ T cells with or without Glutamine supplementation. **b,** CD36 mRNA expression in PBS or RSL3 treated CD8^+^ T cells with or without Glutamine supplementation. N=4 per group. Each symbol represents one individual. Data are mean ± s.e.m.. *p* values are measured by and one-way ANOVA with Tukey’s multiple comparison test. **p*<0.05, ***p*<0.01.


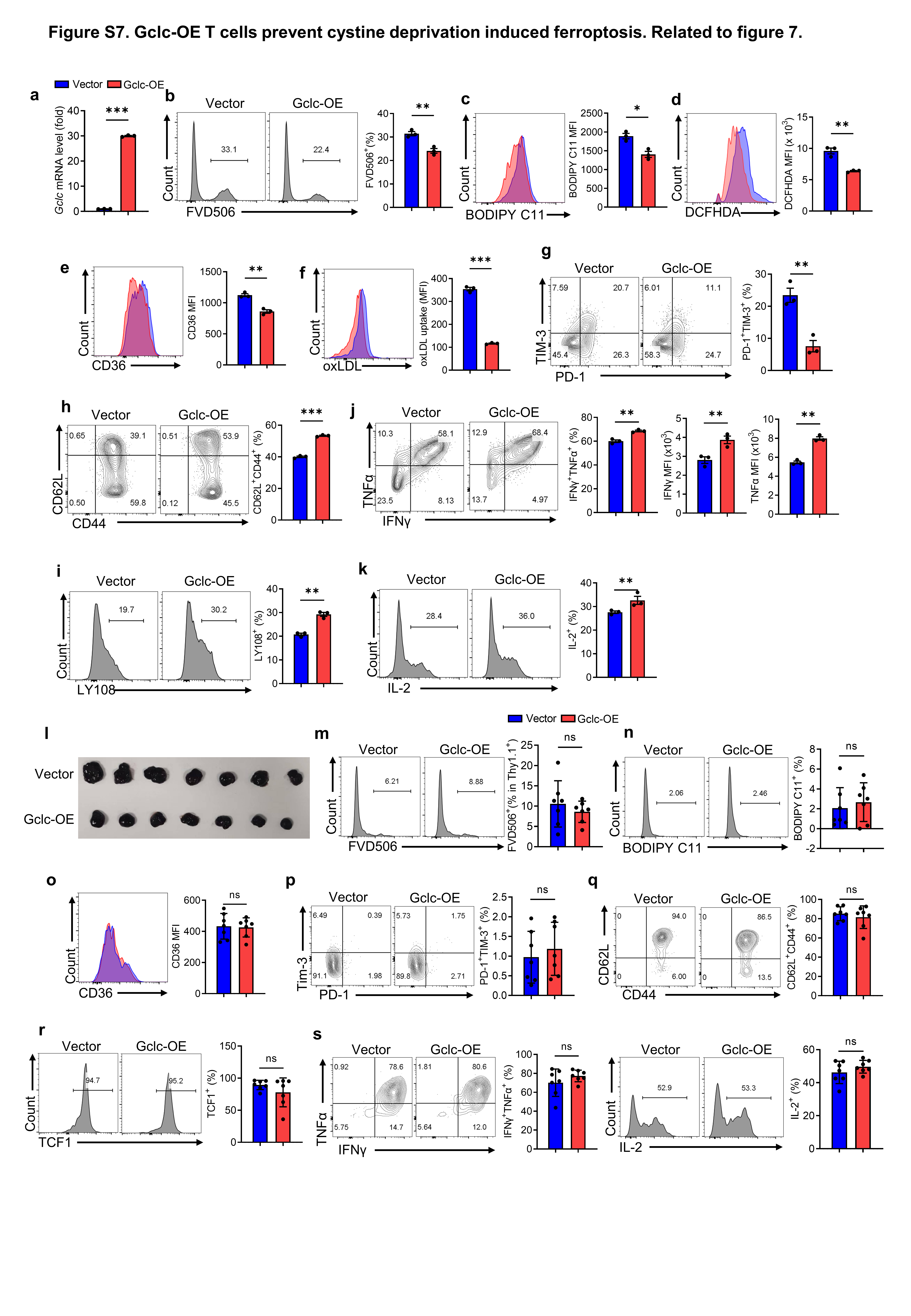


**Supplementary figure 7. Gclc-OE T cells prevent cystine deprivation induced ferroptosis. Related to figure 7.**

**a,** RT-qPCR analysis of *Gclc* expression in control and Gclc-OE T cells (n=3 per group). **b, c,** Vector and Gclc-OE T cells were cultured in CD for 48 hours (n=3 per group), the FVD506^+^ T cells (b) and lipid peroxidation levels (c) were detected by flow cytometry. **d-f** Flow cytometry analysis of DCFHDA MFI (d), CD36 MFI (e), and oxLDL MFI (f) in the indicated T cells. **g-I,** Flow cytometry analysis of the population of PD-1^+^TIM-3^+^ subset (g), CD62L^+^CD44^+^ subset (h), and LY108^+^ subset (i) in the indicated T cells. **j-k,** The levels of IFNγ, TNFα (j), and IL-2 (k) secreted by vector and Gclc-OE T cells. **l,** Image of tumor sizes in the indicated group (n=7 per group). **m, n,** The percentages of dead cells (m) and lipid peroxidation levels (n) of the indicated T cells in spleens (n=7 per group). **o,** CD36 expression of the indicated T cells in spleens. **p-r,** The percentage of the PD-1^+^TIM-3^+^ subset (p), CD62L^+^CD44^+^ subset (q), and TCF1 expression (r) of the indicated T cells in spleens. **s,** The levels of IFNγ, TNFα, and IL-2 secretion the indicated T cells in spleens. Each symbol represents one individual. Data are mean ± s.e.m.. *p* values are measured by two-tailed unpaired Student’s *t* test. ns, not significant, **p*<0.05, ***p*<0.01, ****p*<0.001.

**Supplementary table 1. Antibodies used for FACS and WB analysis**

| **Antibody** | **Source** | **Identififer** |
| --- | --- | --- |
| Brilliant Violet 711-anti-CD8a | Biolegend | Cat# 100748; RRID: AB_2562100 |
| APC anti-mouse CD8a | Biolegend | Cat# 100712; RRID: AB_312751 |
| FITC-anti-CD4 | Biolegend | Cat# 100406; RRID: AB_312691 |
| PE-Cy7-anti-TIM-3 | Invitrogen | Cat# 25-5870-82; RRID: AB_2573483 |
| Percp-eflour 710-anti-PD-1 | Invitrogen | Cat# 46-9981-82; RRID: AB_11151142 |
| APC-anti-CD62L | Biolegend | Cat# 104412; RRID: AB_313099 |
| APC-Cy7-anti-CD44 | Biolegend | Cat# 103028; RRID: AB_830785 |
| APC-anti-LY108 | Biolegend | Cat# 134610; RRID: AB_2728155 |
| Alexa Fluor647-anti-TCF-1 | Cell Signaling Technology | Cat# 6709S; RRID: AB_2797631 |
| PE-TOX | Invitrogen | Cat# 12-6502-82; RRID: AB_10855034 |
| FITC-anti-TNAα | Biolegend | Cat# 506304; RRID: AB_315425 |
| APC-anti-IFNγ | Invitrogen | Cat# 17-7311-82; RRID: AB_469504 |
| PE-IL-2 | Biolegend | Cat# 503808; RRID: AB_315302 |
| Pacific Blue-anti-human/mouse Granzyme B | Biolegend | Cat# 515408; RRID: AB_2562196 |
| APC anti-mouse CD36 | Biolegend | Cat# 102612; RRID: AB_2072639 |
| AF700-anti-Thy1.1(CD90.1) | Biolegend | Cat# 202528; RRID: AB_1626241 |
| APC anti-mouse CD45.1 | Biolegend | Cat# 110714; RRID: AB_313503 |
| PE-anti-CD45.1 | Biolegend | Cat# 110708; RRID: AB_313497 |
| PerCP-Cy5.5-anti-CD45.1 | Biolegend | Cat# 110728; RRID: AB_893346 |
| FITC-anti-CD45.2 | Invitrogen | Cat# 11-0454-85; RRID: AB_465062 |
| PB-anti-CD45.2 | Biolegend | Cat# 109820; RRID: AB_492872 |
| Purified anti-mouse CD36 | Biolegend | Cat# 163002; RRID: AB_2892336 |
| Purified anti-mouse CD8a | Biolegend | Cat# 100702; RRID: AB_312741 |
| Anti-Mo CD3e | Invitrogen | Cat# 16-0031-86;  RRID: AB_468849 |
| Anti-Mo CD28 | Invitrogen | Cat# 16-0281-85;  RRID: AB_468922 |
| GCLC Polyclonal Antibody | Abclonal | Cat# A1038; RRID: AB_2757927 |
| SLC7A11 / xCT Rabbit mAb | Abclonal | Cat# A2413; RRID: AB_2863004 |
| beta Actin antibody | Abcam | Cat# ab8227; RRID: AB_2305186 |
| Goat anti-mouse IgG-HRP | Absin Bioscience | Cat# abs20001A; RRID: AB_2716555 |
| Goat anti-rabbit IgG-HRP | Absin Bioscience | Cat# abs20002A; RRID: AB_2716554 |

**Supplementary table 2. Forward and reverse primers for RT-qPCR.**

| Gene | Forward | Reverse |
| --- | --- | --- |
| *Slc7a11* | AGGGCATACTCCAGAACACG | GGACCAAAGACCTCCAGAATG |
| *Gclc* | GGGGTGACGAGGTGGAGTA | GTTGGGGTTTGTCCTCTCCC |
| *Cd36* | ATGGGCTGTGATCGGAACTG | TTTGCCACGTCATCTGGGTTT |
| *Actb* | GGCTGTATTCCCCTCCATCG | CCAGTTGGTAACAATGCCATGT |
