# Supplementary figures and images for: Cystine deprivation triggers CD36-mediated ferroptosis and dysfunction of tumor infiltrating CD8+ T cells
Source: Cell Death Dis. 2024 Feb 15;15(2):145. doi: 10.1038/s41419-024-06503-1 (PMC10869360; doi:10.1038/s41419-024-06503-1)

Supplementary File Uncropped WB


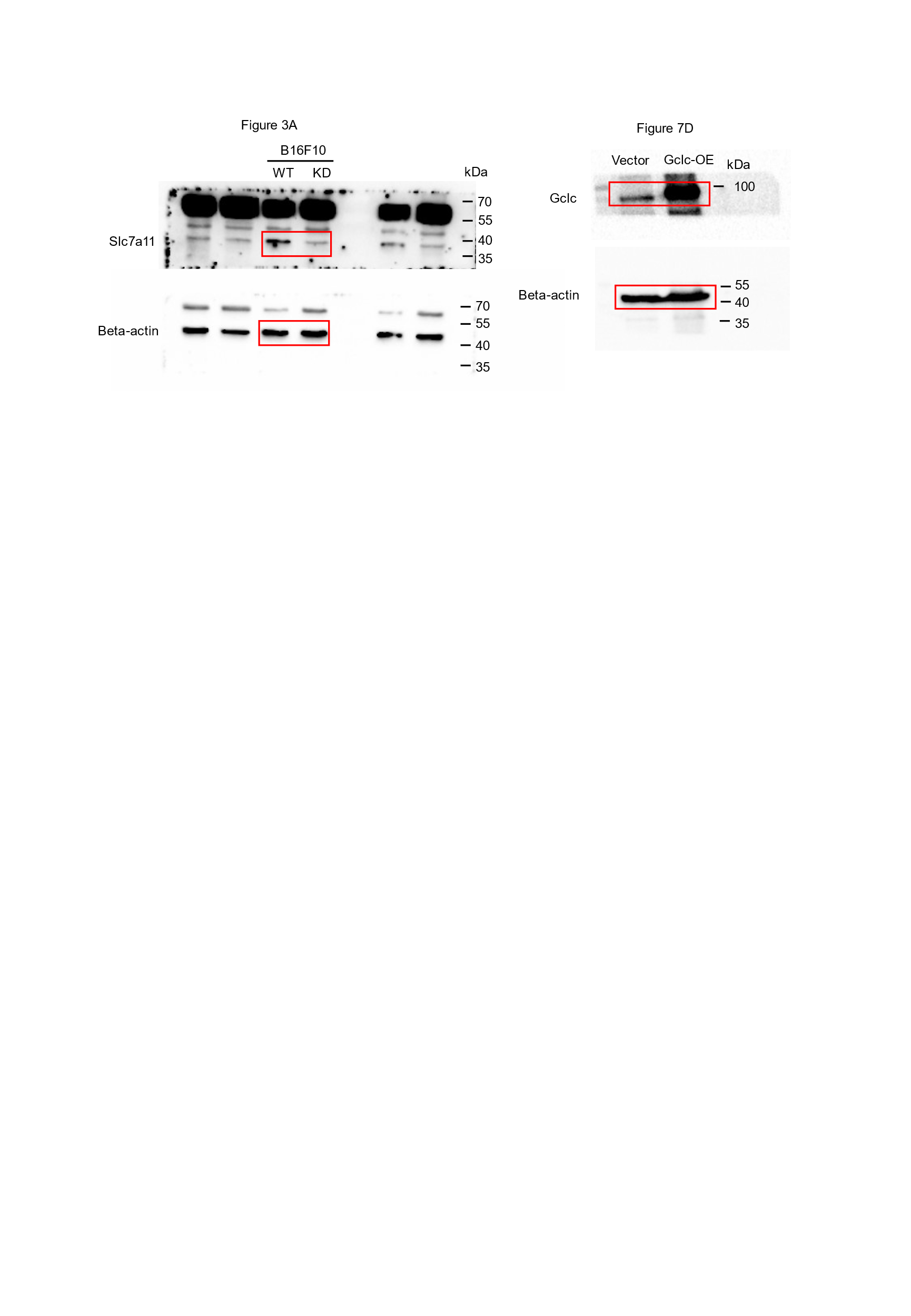

Supplement: Supplementary file 2 — Supplementary File Uncropped WB [file 41419_2024_6503_MOESM2_ESM.docx]
